# A family of homologous metal heteromaterials with chemically bonded metallic interface

**DOI:** 10.1093/nsr/nwaf311

**Published:** 2025-08-04

**Authors:** Heming Liu, Qiangmin Yu, Jiarong Liu, Huang Chen, Yumo Chen, Tianhao Zhang, Jahangir Khan, Yuxiao Dong, Xin Kang, Le Liu, Hui-Ming Cheng, Bilu Liu

**Affiliations:** Shenzhen Geim Graphene Center, Shenzhen Key Laboratory of Advanced Layered Materials for Value-added Applications, Key Laboratory of Electrocatalytic Materials and Green Hydrogen Technology of Guangdong Higher Education Institutes, Institute of Materials Research, Tsinghua Shenzhen International Graduate School, Tsinghua University, Shenzhen 518055, China; Shenzhen Geim Graphene Center, Shenzhen Key Laboratory of Advanced Layered Materials for Value-added Applications, Key Laboratory of Electrocatalytic Materials and Green Hydrogen Technology of Guangdong Higher Education Institutes, Institute of Materials Research, Tsinghua Shenzhen International Graduate School, Tsinghua University, Shenzhen 518055, China; Shenzhen Geim Graphene Center, Shenzhen Key Laboratory of Advanced Layered Materials for Value-added Applications, Key Laboratory of Electrocatalytic Materials and Green Hydrogen Technology of Guangdong Higher Education Institutes, Institute of Materials Research, Tsinghua Shenzhen International Graduate School, Tsinghua University, Shenzhen 518055, China; Shenzhen Geim Graphene Center, Shenzhen Key Laboratory of Advanced Layered Materials for Value-added Applications, Key Laboratory of Electrocatalytic Materials and Green Hydrogen Technology of Guangdong Higher Education Institutes, Institute of Materials Research, Tsinghua Shenzhen International Graduate School, Tsinghua University, Shenzhen 518055, China; Shenzhen Geim Graphene Center, Shenzhen Key Laboratory of Advanced Layered Materials for Value-added Applications, Key Laboratory of Electrocatalytic Materials and Green Hydrogen Technology of Guangdong Higher Education Institutes, Institute of Materials Research, Tsinghua Shenzhen International Graduate School, Tsinghua University, Shenzhen 518055, China; Shenzhen Geim Graphene Center, Shenzhen Key Laboratory of Advanced Layered Materials for Value-added Applications, Key Laboratory of Electrocatalytic Materials and Green Hydrogen Technology of Guangdong Higher Education Institutes, Institute of Materials Research, Tsinghua Shenzhen International Graduate School, Tsinghua University, Shenzhen 518055, China; Shenzhen Geim Graphene Center, Shenzhen Key Laboratory of Advanced Layered Materials for Value-added Applications, Key Laboratory of Electrocatalytic Materials and Green Hydrogen Technology of Guangdong Higher Education Institutes, Institute of Materials Research, Tsinghua Shenzhen International Graduate School, Tsinghua University, Shenzhen 518055, China; Shenzhen Geim Graphene Center, Shenzhen Key Laboratory of Advanced Layered Materials for Value-added Applications, Key Laboratory of Electrocatalytic Materials and Green Hydrogen Technology of Guangdong Higher Education Institutes, Institute of Materials Research, Tsinghua Shenzhen International Graduate School, Tsinghua University, Shenzhen 518055, China; Shenzhen Geim Graphene Center, Shenzhen Key Laboratory of Advanced Layered Materials for Value-added Applications, Key Laboratory of Electrocatalytic Materials and Green Hydrogen Technology of Guangdong Higher Education Institutes, Institute of Materials Research, Tsinghua Shenzhen International Graduate School, Tsinghua University, Shenzhen 518055, China; Shenzhen Geim Graphene Center, Shenzhen Key Laboratory of Advanced Layered Materials for Value-added Applications, Key Laboratory of Electrocatalytic Materials and Green Hydrogen Technology of Guangdong Higher Education Institutes, Institute of Materials Research, Tsinghua Shenzhen International Graduate School, Tsinghua University, Shenzhen 518055, China; Shenyang National Laboratory for Materials Science, Institute of Metal Research, Chinese Academy of Sciences, Shenyang 110016, China; Institute of Technology for Carbon Neutrality, Shenzhen Institute of Advanced Technology, Chinese Academy of Sciences, Shenzhen 518055, China; Shenzhen Geim Graphene Center, Shenzhen Key Laboratory of Advanced Layered Materials for Value-added Applications, Key Laboratory of Electrocatalytic Materials and Green Hydrogen Technology of Guangdong Higher Education Institutes, Institute of Materials Research, Tsinghua Shenzhen International Graduate School, Tsinghua University, Shenzhen 518055, China

**Keywords:** homologous metal heteromaterials, interface bonding, interface conductivity, high-throughput screening, high-current-density water splitting

## Abstract

Heteromaterials made of two different components have been widely studied; their interfacial bonding is essential to control their properties. How to prepare a chemically bonded metallic interface remains elusive, despite its importance for structural and functional materials. Here we synthesized a family of homologous metal heteromaterials (HMHs) featuring chemically bonded metallic interfaces, which endow them with strong interfacial binding forces and metallic conductivity. These features stem from delocalized electronic states and a uniformly distributed electric field across the interface of HMHs. We synthesized and high-throughput screened a family of HMHs comprising four categories and 20 materials. As an application example, we show that HMHs operate stably in a water electrolyzer with a decay rate of 1.06 μV h^−1^ at high current density over 1000 h, thanks to the above two interface properties. To our knowledge, this is the lowest decay rate reported to date, surpassing the target of the U.S. Department of Energy for 2040.

## Teaser

A new family of homologous metal heteromaterials featuring chemically bonded and metallic interface achieves high performance water electrolysis at high current density, surpassing the 2040 target of the U.S. Department of Energy.

## INTRODUCTION

Integrating dissimilar substances is effective to develop new material structures and functions in interfacial science [1], electronics [2], electron microscopy [3], material design [4,5], etc. In such systems, bonding between the two components is key to control interfacial properties and even the properties of the whole materials. Two typical interface bonding types are van der Waals (vdW) interaction and chemical bonding. The vdW interaction is weak and provides an atomically flat interface with few defect states, which is advantageous for semiconductor electronics and optoelectronics [6–8]. Chemical bonds typically exhibit bonding strength 2–3 orders of magnitude higher than vdW interactions [9], and can regulate the mechanical properties and electronic structures of heteromaterials [10,11]. Regulating the interfacial bonding is crucial for achieving designed functions of materials.

A chemically bonded metallic interface is essential for materials that operate under extreme conditions and require good conductivity. On the one hand, the strong chemical bonds at the heterointerface can improve mechanical properties of materials such as stiffness and fatigue resistance [12,13]. On the other hand, a low interfacial resistance can reduce energy consumption and enhance energy efficiency of electronics and energy devices [14,15]. The above two factors are particularly important for electrochemical gas evolution reactions at high current densities. First, the vdW interaction energy (0.1–1 J m^−2^) at the interface of a heterostructure catalyst is insufficient to withstand large bubble adhesion energy (1–100 J m^−2^) at high current densities [16,17]. Second, the charge transfer barrier and defects at the interface in common heterostructure materials cause sluggish charge transfer kinetics [18–20]. Therefore, two criteria including both strong interfacial binding and metallic conductivity are prerequisites for heterostructure catalysts at high current densities, yet this is challenging. As a type of heterostructure catalysts, metal and metal compound heterostructures are widely explored. However, due to differences in electronic and crystal structures between metals and metal compounds, it is difficult to simultaneously achieve the above two properties in such heterostructures. Existing methods such as wet chemical synthesis [21,22] and vapor deposition [23,24] provide poor conductivity or weak adhesion between metal substrates and metal compounds [25]. Other methods like heating under a reducing atmosphere can generate strong chemical bonds between metals and metal oxide supports. Nevertheless, the strong metal–support interaction may enable metal nanoparticles to be largely covered by suboxide species, which will create a Schottky barrier at the interface to impede electron transfer [18,26–28]. Therefore, how to prepare a chemically bonded metallic interface in heterostructures remains challenging.

**Figure 1.**
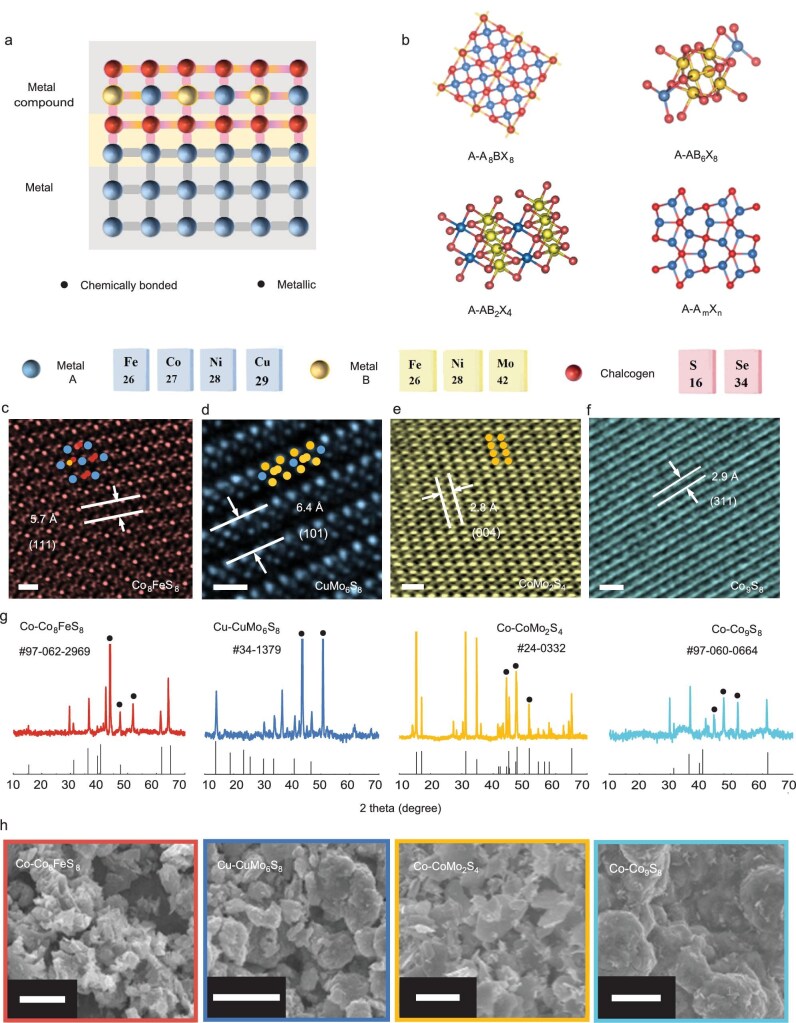
Synthesis and characterization of a family of HMHs. (a) Schematic of HMHs with chemically bonded metallic interface. (b) Atomic models of four types of HMHs. (c–f) High-resolution transmission electron microscopy (HRTEM) images of four types of HMHs. The scale bars are 0.5 nm. (g and h) XRD patterns and scanning electron microscopy (SEM) images of four types of HMHs. The scale bars are 2 μm. The peaks in XRD patterns marked by filled circles are from metal substrates.

Here, we developed an interfacial bonding strategy to prepare a family of homologous metal heteromaterials (HMHs) featuring a chemically bonded metallic interface. HMHs have a strong interfacial binding force at the interface to maintain high mechanical stability, and a metallic interface to eliminate the charge transfer barrier (Fig. 1a). Delocalized electronic states and a uniformly distributed electric field at the interface confirm the formation of interfacial chemical bonds and metallic property. We achieved universal synthesis of HMHs by metal source diffusion into a precursor to obtain four categories and 20 materials. We also achieved scalable production and high-throughput screening of HMHs using an *in situ* optical polarization imaging method. Exemplified by electrocatalysis application, HMHs operate stably in an anion exchange membrane water electrolyzer (AEMWE) with a decay rate of 1.06 μV h^−1^ at a high current density of 500 mA cm^−2^ over 1000 h. To our knowledge, the decay rate is the lowest reported to date at high current densities, and surpasses the target of the U.S. Department of Energy (DOE) for 2040.

## RESULTS

### Synthesis and characterization of a family of HMHs

The HMHs were prepared by a metal source diffusion-initiated interfacial bonding method (see details in Methods). We chose metal A (A = Fe, Co, Ni, Cu) and metal chalcogenide BX_2_ (B = Fe, Ni, Mo; X = S, Se) as substrates and precursors to prepare a family of HMHs including four categories (A–A_8_BX_8_, A–AB_6_X_8_, A–AB_2_X_4_ and A–A_m_X_n_) and 20 materials (Fig. 1b, Figs S1–S3 and Table S1). Among these, the A–A_8_BX_8_ materials have a cubic structure consisting of A–X octahedrons and B–X tetrahedrons. The A–AB_6_X_8_ materials crystallize in a rhombohedral symmetry with the *R*-3 space group. The A–AB_2_X_4_ materials feature monoclinic structures composed of A–X and B–X octahedrons in an alternating stacking arrangement. The A–A_m_X_n_ materials contain six kinds of monometallic chalcogenides, which are synthesized from chalcogen elements. The representatives of the above four categories include cobalt iron sulfide (Co_8_FeS_8_), copper molybdenum sulfide (CuMo_6_S_8_), cobalt molybdenum sulfide (CoMo_2_S_4_) and cobalt sulfide (Co_9_S_8_) (Fig. 1c–h, Figs S4 and S5). All these materials have zero band gaps and metallic properties, forming all-metallic heterostructures.

### Chemically bonded metallic interface in HMHs

**Figure 2.**
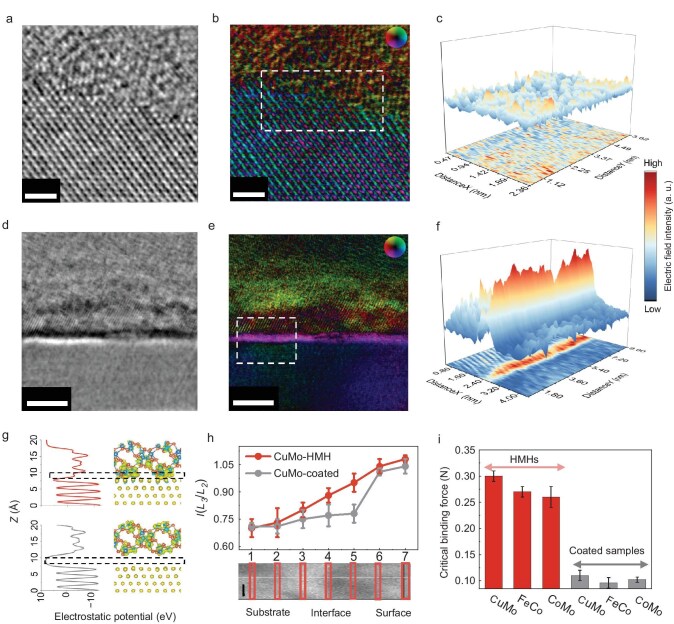
Chemically bonded metallic interface in HMHs. (a–c) Integral differential phase contrast-scanning transmission electron microscopy (iDPC-STEM), DPC-STEM and corresponding 3D mappings of electric field intensity of CuMo HMH. The scale bars are 1 nm. (d–f) iDPC-STEM, DPC-STEM and corresponding 3D mappings of electric field intensity of CuMo-coated sample. The scale bars are 5 nm. (g) The electrostatic potentials and differential charge distributions at the interfaces of CuMo-HMHs (upper) and CuMo-coated samples (bottom). Yellow, blue and red atoms represent Cu, Mo and S atoms, respectively. Blue and yellow areas indicate the loss and accumulation of electrons. (h) Intensity ratio between *L*_3_ and *L*_2_ edge [*I*(*L*_3_/*L*_2_)] of Cu elements of CuMo-HMHs and CuMo-coated samples from substrate to surface in annular dark field image. The scale bar is 2 nm. (i) Comparison of critical binding forces of HMHs and coated samples.

We used scanning transmission electron microscopy (STEM) to characterize interfacial atomic structures of HMHs and coated samples, which were used as control samples. HMHs were made by spraying metal chalcogenides onto the metal substrate, followed by annealing at high temperature. Coated samples were made by spray coating but without annealing. For HMHs, exemplified by the CuMo HMH, we sprayed MoS_2_ onto Cu substrate and annealed them at high temperature (see Methods for details). Cu atoms diffuse from substrate to surface at high temperature, resulting in an interface between Cu and CuMo_6_S_8_ with little lattice distortion. The interplanar spacings of Cu and CuMo_6_S_8_ are 0.21 and 0.44 nm, corresponding to (11-1) and (012) planes (Fig. S6). In contrast, there are obvious lattice mismatches and vdW gaps at the interface of the coated sample made by spray coating CuMo_6_S_8_ on Cu substrate at room temperature (Fig. S7). The integral differential phase contrast (iDPC) and DPC images of STEM indicate uniform intensities of electrostatic potential and electric field in the two regions of CuMo-HMH (Fig. 2a and b) [29,30]. The intensity of the local electric field at the interface is nearly six times higher than that in two regions of the coated sample (Fig. 2c–f). The calculated electrostatic potentials and differential charge distributions further confirm remarkable electronic interaction and delocalized electronic state at the interface of CuMo-HMH, which is attributed to the formation of chemical bonds (Fig. 2g, Fig. S8). However, a localized electronic state without electronic interaction is observed at the interface of the coated sample (Fig. 2g). Taken together, we confirmed that the formation of chemical bonds in metallic materials can eliminate the interfacial charge transfer barrier in CuMo-HMHs.

We then applied electron energy loss spectroscopy (EELS) of Cu *L*_3_ and *L*_2_ edges to analyze bonding properties. The valence state of Cu can be analyzed by the intensity ratio between *L*_3_ and *L*_2_ edges [*I*(*L*_3_/*L*_2_)] and *L*_3_ edge position [31]. The results show a gradient change of *I*(*L*_3_/*L*_2_) from Cu (0.70) to CuMo_6_S_8_ (1.08) in the CuMo-HMH, indicating that the valence state of Cu increases from 0 to +1 or higher, and the existence of chemical bonds at the interface. In contrast, the *I*(*L*_3_/*L*_2_) ratio shows negligible change at the interface of the CuMo-coated sample, which is attributed to weak interaction or vdW gaps at the interface (Fig. 2h and Fig. S9). We observed similar phenomena in other HMHs and corresponding coated samples, confirming the universality of the interfacial property (Figs S10–S15). Micro-scratch experiments show that the critical binding forces of HMHs are 2–3 times higher than those of coated samples, further confirming that HMHs with interfacial chemical bonds have higher mechanical stability than coated samples with weak interaction at the interface (Fig. 2i, Figs S16 and S17). The above results demonstrate that HMHs have a strong chemical bonded interface with barrier-free charge transfer.

### Scalable preparation and high-throughput catalytic activity screening of HMHs

**Figure 3.**
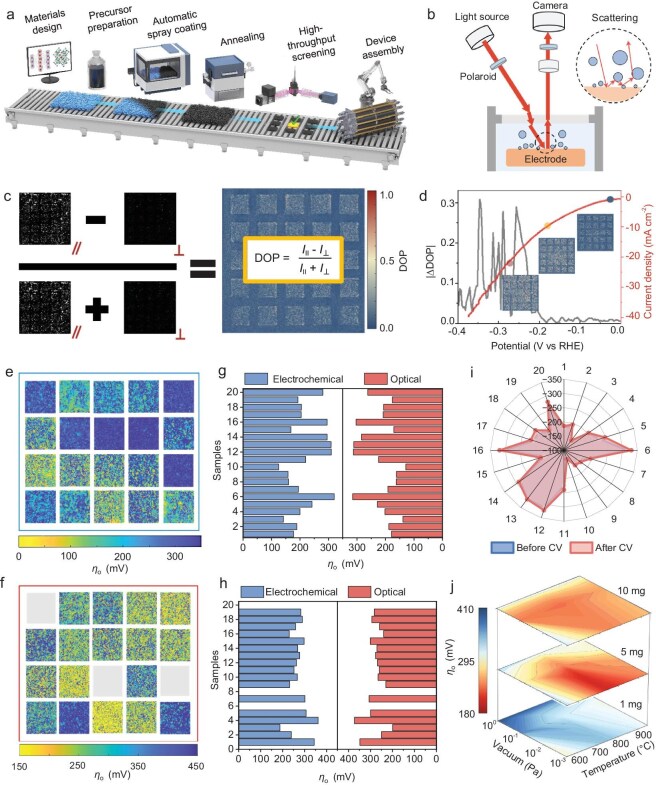
Scalable preparation and high-throughput activity screening of HMHs. (a) Schematic of scalable preparation of HMHs. (b) Test principle of *in situ* polarization imaging method. (c) The DOP distribution of 20 HMHs in a single frame recorded by camera. (d) Plots of |ΔDOP| intensity changing with applied bias (left) and corresponding linear scanning voltammetry curve (right). RHE, reversible hydrogen electrode. (e and f) Mappings of onset overpotentials (*η*_o_) of 20 HMHs for HER (e) and OER (f). (g and h) Optical screening *η*_o_ values and electrochemical *η*_o_ values for HER (g) and OER (h). (i) Optical screening *η*_o_ values of 20 HMHs for HER before and after 1000 CVs. (j) Optical screening *η*_o_ mappings of CoFe-HMHs for OER under different synthesis conditions.

HMHs are promising electrocatalysts given their interfacial properties mentioned above. We developed a flow production line to prepare large-area HMH-based electrodes with sizes of 10 × 10 cm^2^, and then high-throughput screened their catalytic activity using an *in situ* optical polarization imaging method (see details in Methods, Fig. 3a and Fig. S18). The method is based on changes in the degree of polarization (DOP) when the polarized light is scattered by tiny bubbles [32,33] (Fig. 3b and Fig. S19). The DOP distribution of 20 HMHs in a single frame is shown in Fig. 3c. The overpotential corresponding to the point of |ΔDOP| mutation is defined as the onset overpotential (*η*_o_) to reflect intrinsic activity of HMHs (Fig. 3d and Supplementary Video). The consistency of experimental results confirm that the method is reliable and reproducible (Figs S20 and S21). The optical screening results of the hydrogen evolution reaction (HER) and oxygen evolution reaction (OER) activities of 20 HMH electrodes are shown in Fig. 3e–h. As exemplified by the OER results, CoFe-HMH exhibits the highest activity with *η*_o_ < 200 mV. Nine other HMHs show moderate activity with *η*_o_ between 200 and 300 mV, while the remaining HMHs show poor activity with *η*_o_ > 300 mV (Fig. 3e–h and Table S2). The optical screening results are close to those obtained by electrochemical tests, demonstrating the reliability of this method (Fig. 3g and h). We further evaluated the stability of 20 HMHs by comparing *η*_o_ before and after 1000 cyclic voltammetry (CV). The differences of *η*_o_ between optical and electrochemical methods for most samples were <15 mV, indicating the reliability of the *in situ* polarization imaging method (Fig. 3i, Figs S22 and S23). We further optimized synthesis conditions for CoFe-HMHs, finding that the optimized conditions include a mass loading of 5 mg cm^−2^, an annealing temperature of 750°C, and a vacuum of 10^−3^ Pa (Fig. 3j, Fig. S24 and Table S3). Taken together, we achieved scalable production and high-throughput screening of catalytic activity, stability and synthesis conditions of the HMHs family using an *in situ* polarization imaging method.

### Application of HMHs in high-current-density electrocatalysis

**Figure 4.**
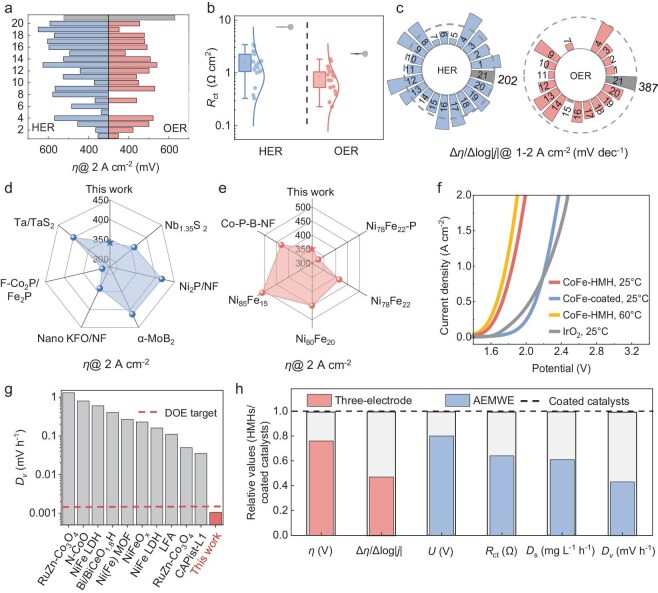
Application of HMHs in high-current-density electrocatalysis. (a) Overpotentials (*η*) at 2 A cm^−2^, (b) charge transfer resistances (*R*_ct_) and (c) Δ*η*/Δlog|*j*| at 1–2 A cm^−2^ of 20 HMHs for HER and OER. The gray ones represent the Pt/C (left) and IrO_2_ (right) catalysts. The numbered sample labels from 1 to 20 correspond to Co–Co_8_FeS_8_, Co–NiCo_8_S_8_, Co–Co_9_S_8_, CoNi–Co_9_S_8_, Cu–CuMo_6_S_8_, Cu–CuMo_2_S_3_, Fe–FeMo_6_S_8_, Cu–CuMo_6_Se_8_, Fe–FeMo_6_Se_8_, Co–CoMo_2_S_4_, Ni–FeNi_2_S_4_, Fe–FeMo_2_S_4_, Ni–NiCo_2_Se_4_, Fe–CoFe_2_Se_4_, Ni–Ni_3_S_2_, Fe–FeS, Fe–FeSe, Mo–Mo_15_Se_19_, Ni–Ni_3_Se_2_ and Co–Co_3_Se_4_, respectively. (d and e) Comparisons of *η* at 2 A cm^−2^ of CuMo-HMHs (HER) and CuFe-HMHs (OER) with that in other literature. (f) Linear scanning voltammetry (LSV) curves of HMHs, coated catalysts and IrO_2_-based AEMWEs. (g) Comparisons of decay rate (*D*_v_) of HMH-based AEMWE with that in other literature. (h) Values of HMHs relative to coated catalysts in terms of *η* at 2 A cm^−2^, Δ*η*/Δlog|*j*| at 1–2 A cm^−2^, potential (*U*) at 2 A cm^−2^, *R*_ct_, dissolution rate of Fe and *D*_v_. The dashed line represents the reference value of coated catalysts.

High-current-density electrocatalysis is important for industrial applications but poses strict requirements for the mechanical stability and electron transfer of electrocatalysts. We have tested HER, OER and nitrate reduction reaction performance of HMH electrodes at high current density (Fig. S25). Taking water electrolysis as an example, for HER, half of the HMHs have lower overpotentials (*η*) than that of the commercial Pt/C catalyst at 2 A cm^−2^. For OER, almost all HMHs have lower *η* than that of the commercial IrO_2_ catalyst at 2 A cm^−2^ (Fig. 4a and Table S2). The charge transfer resistance (*R*_ct_) and the indicator Δ*η*/Δlog|*j*| demonstrate that HMHs possess faster charge transfer kinetics, lower bubble adhesion force and better mass transfer than those of Pt/C, IrO_2_ and other coated catalysts (Fig. 4b and c and Figs S26–S31). This is because, first, HMHs have delocalized electronic states at the metallic interface, eliminating the interfacial electric field barrier and improving the electron transfer efficiency at high current density. Second, the good mechanical stability of HMHs is attributed to the strong chemical bonding at the interface, which allows surface catalysts to be firmly anchored to the substrate without falling off due to bubble adhesion. The high surface energy of metallic HMHs makes the surface highly hydrophilic, which promotes rapid bubble evolution and reduces bubble adhesion force. In addition, the high intrinsic activity of the non-metal sites of metallic compounds, combined with the good conductivity of the metallic substrate, together give HMHs high performance at high current density. Among the tested HMHs, CuMo-HMH and CoFe-HMH show the best HER and OER performance at 2 A cm^−2^ with *η* of 336 and 349 mV, surpassing the performance in most literature (Figs 4d and e and Tables S4 and S5). We further assembled an AEMWE with HMHs, and it needed 1.76 V at 1 A cm^−2^ and 1.89 V at 2 A cm^−2^, better than AEMWEs made of IrO_2_ and other coated catalysts (Fig. 4f). The improved electrolyzer performance is attributed to the low interfacial resistance, fast mass transfer and bubble evolution kinetics of HMHs (Fig. S32). The HMH-based AEMWE works stably at high current density of 500 mA cm^−2^ over 1000 h with a decay rate (*D*_v_) of 1.06 μV h^−1^. To our best knowledge, this is the lowest decay rate reported to date (Fig. 4g, Fig. S33 and Table S6) and surpasses the 2040 target of the U.S. DOE [34]. The charge transfer efficiency (Δ*η*/Δlog|*j*| at 1–2 A cm^−2^ and *R*_ct_), apparent performance (*η* and potential at 2 A cm^−2^) and stability (*D*_v_ and dissolution rate of Fe) of HMHs are more than 2, 1.25 and 2 times those of coated catalysts (Fig. 4h). We also conducted accelerating stress tests (ASTs) on both electrodes and electrolyzers under harsh working conditions. The HMHs showed negligible catalyst falling-off, and corresponding AEMWE showed a lower *D*_v_ and dissolution rate of Fe than that of coated catalysts during ASTs (Figs S34–S36). Given the unique interfacial mechanical and electrical properties of HMHs, the AEMWE demonstrates superior performance and stability compared to coated catalysts at high current density and during ASTs.

## DISCUSSION

In summary, we synthesized a family of HMHs featuring strongly chemically bonded metallic interfaces. Delocalized electronic states at the interface and uniformly distributed interfacial electric field are responsible for the above two features. We carried out universal synthesis of HMHs and obtained four categories and 20 materials, enabling scalable production and high-throughput screening of HMHs by using the *in situ* polarization imaging method. As an example, the water electrolyzer using HMHs works stably at 500 mA cm^−2^ over 1000 h with a decay rate of 1.06 μV h^−1^, which is the lowest reported to date at high current density, and surpasses the 2040 target of the U.S. DOE. The water electrolyzer also works stably during ASTs. This work paves the way for constructing heterointerfaces and understanding new interface conductance mechanisms, and also provides potential solutions for addressing interface electrical contact issues in electronics and energy devices.

## MATERIALS AND METHODS

### Preparation of homologous metal heteromaterials

All chemicals were utilized without further purification. Metal foams (Kunshan Jiayisheng Electronics Co., Ltd., China) were cut into 1 × 1.5 cm^2^ pieces. First, we subjected metal foams to sonication in a 1 M HCl aqueous solution for 30 min followed by washing them with deionized water. Second, we put the metal chalcogenide precursor into a mortar grinder (Retsch RM 200, Germany) to obtain a fine powder [35]. We then dispersed the powder into ethanol and kept it under sonication for 30 min to make a well-dispersed solution. After this, we dropped the dispersion onto metal foams to reach a mass loading of 5 mg cm^−2^. Subsequently, we placed samples in a glass tube and evacuated to 10^−3^ Pa. Finally, we annealed the vacuum-sealed glass tube in a furnace according to the required temperature and time conditions. Under high-temperature annealing, the metal chalcogenide precursor reacted with the metal substrate to form a heterostructure. Some materials were annealed under conditions of Ar (200 standard cubic cm/min, sccm) and H_2_ (10 sccm) without vacuum environment. The synthesis details are shown in Table S1.

### Material characterizations

Phase and crystal structure of samples were characterized by X-ray diffraction (XRD, Rigaku, SmartLab 9KW, Japan) with a scan rate of 10° min^−1^ from 10° to 90°. The surface morphologies were characterized by field-emission scanning electron microscopy (FE-SEM, ZEISS Sigma300, USA) with an accelerated voltage of 10 kV. Atomic structures and element distribution were characterized by spherical aberration-corrected STEM (Thermo Scientific Spectra 300, USA) with an accelerating voltage of 300 kV. Interfacial electric field and electrostatic potential in samples were characterized by differential phase contrast (DPC) and integral DPC modes of STEM. Chemical analysis was performed by high resolution X-ray photoelectron spectroscopy (XPS, Thermo Fisher ESCALAB 250Xi, USA). The critical binding force was characterized by micro scratch tester (Anton Paar, UNHT, Austria). The contact angle was tested by contact angle meter (KRUSS DSA30, Germany). The bubble adhesion force was tested by high-sensitivity micro-electrochemical balance (DataPhysics DCAT 21, Germany). The concentration of ion dissolved in electrolyte was tested by inductively coupled plasma-optical emission spectroscopy (ICP-OES, Spectro Arcos II MV, Germany).

## Supplementary Material

nwaf311_Supplemental_Files
