# Supplementary material for: A family of homologous metal heteromaterials with chemically bonded metallic interface
Source: Natl Sci Rev. 2025 Aug 4;12(9):nwaf311. doi: 10.1093/nsr/nwaf311 (PMC12400802; doi:10.1093/nsr/nwaf311)
Supplement: nwaf311_Supplemental_Files [file nwaf311_supplemental_files.zip › Supplementary data.pdf]

Supplementary Materials for National Science Review

A family of homologous metal heteromaterials with chemically bonded metallic interface

Heming Liu[1,#], Qiangmin Yu[1,#], Jiarong Liu[1], Huang Chen[1], Yumo Chen[1], Tianhao Zhang[1], Jahangir Khan[1], Yuxiao Dong[1], Xin Kang[1], Le Liu[1], Hui-Ming Cheng[2,3] and Bilu Liu[1,*]

1. Shenzhen Geim Graphene Center, Shenzhen Key Laboratory of Advanced Layered Materials for Value-added Applications, Key Laboratory of Electrocatalytic Materials and Green Hydrogen Technology of Guangdong Higher Education Institutes, Institute of Materials Research, Tsinghua Shenzhen International Graduate School, Tsinghua University, Shenzhen 518055, China;
2. Shenyang National Laboratory for Materials Science, Institute of Metal Research, Chinese Academy of Sciences, Shenyang 110016, China;
3. Institute of Technology for Carbon Neutrality, Shenzhen Institute of Advanced Technology, Chinese Academy of Sciences, Shenzhen 518055, China

***Corresponding author**. E-mail: bilu.liu@sz.tsinghua.edu.cn

[#]Equally contributed to this work.

This PDF file includes:

- Supplementary Figs S1-S37
- Supplementary Tables S1-S7
- References

Table of Contents

Materials and Methods 4

Supplementary Figures 9

Fig. S1 Schematic of HMHs synthesis. 9

Fig. S2 Phase characterization of other HMHs. 9

Fig. S3 Morphology characterization of other HMHs. 10

Fig. S4 XPS spectra of HMHs. 11

Fig. S5 SEM images of HMHs after OER. 12

Fig. S6 HRTEM images and corresponding FFT patterns of CuMo-HMH. 12

Fig. S7 HRTEM images and corresponding FFT patterns of CuMo-coated sample. 13

Fig. S8 DFT calculation results of three types of HMHs. 13

Fig. S9 EELS spectra of Cu *L* edge of CuMo-HMH and CuMo-coated sample. 14

Fig. S10 HRTEM images and corresponding FFT patterns of CoFe-HMH. 14

Fig. S11 DPC-STEM images of CoFe-HMH. 15

Fig. S12 EELS spectra of Fe and Co *L* edges of CoFe-HMH. 15

Fig. S13 EELS spectra of Fe and Co *L* edges of CoFe-coated sample. 16

Fig. S14 DPC-STEM images of CoMo-HMH. 16

Fig. S15 EELS spectra of Co *L* edges of CoMo-HMH and CoMo-coated sample. 17

Fig. S16 Micro-scratch mechanical experiments of HMHs. 177

Fig. S17 Micro-scratch mechanical experiments of coated samples. 18

Fig. S18 Large-area HMH electrodes with dimensions of 10 × 10 cm^2. 19

Fig. S19 Schematics of in situ polarization imaging technology setups. 19

Fig. S20 Consistency experimental results of Ni foams. 20

Fig. S21 Reproducibility and error analysis of multiple batches. 20

Fig. S22 Mappings of onset overpotentials of 20 HMHs for HER. 21

Fig. S23 Differential mappings of HER overpotentials of 20 HMHs before and after CV. 21

Fig. S24 Optical screening results of 27 synthesis conditions of CoFe-HMH. 21

Fig. S25 Optical screening results of 27 synthesis conditions of CoFe-HMH. 22

Fig. S26 Electrochemical test results of CuMo-HMH, CuMo-coated and MoS2-coated samples. 23
Fig. S27 Electrochemical test results of A-A_8BX_8. 24
Fig. S28 Electrochemical test results of A-A_8BX_8. 24
Fig. S29 Electrochemical test results of A-AB_2X_4. 25
Fig. S30 Electrochemical test results of A-AB_xX_y. 25
Fig. S31 Bubble adhesion force results of HMHs and commercial catalysts. 26
Fig. S32 Electrochemical test results of CuMo-HMH, CuMo-coated and MoS_2-coated catalysts. 27
Fig. S33 HMHs based AEMWE........318
Fig. S34 AST results of HMHs in three-electrode system........319
Fig. S35 Electrochemical test results of HMHs and coated catalysts in AEMWE. 30
Fig. S36 SEM images of HMH and coated catalyst electrodes in AEMWE after AST. 30
Fig. S37 |ΔDOP|-potential curve........31
Supplementary Table 32
Table S1. Synthesis conditions of HMHs. 33
Table S2. Electrochemical parameters of HMHs and references. 34
Table S3. Optical and electrochemical screening results of CoFe-HMH 35
Table S4. Comparison of HER performance between CuMo-HMH and other reported non-noble metal catalysts at 2 A cm^{-2}. 35
Table S5. Comparison of OER performance between CoFe-HMH and other reported non-noble metal catalysts at 2 A cm^{-2}. 35
Table S6. Comparison of AEMWE stability between HMHs and other reported devices running for over hundreds of hours. 36
Table S7. Volume parameter for Co-Co_8FeS_8, Cu-$CuMo_6S_8$, Co-$CoMo_2S_4$ HMHs and corresponding mismatch degrees........37
References 38

Materials and Methods

Electrochemical measurements in three-electrode system

We conducted electrochemical measurements by an electrochemical workstation with amplifier (Zahner Pro, Germany). In all tests, 1 M KOH solution was used as electrolyte. We used the Hg/HgO electrode and IrO_2 electrode as reference and counter electrodes in a standard three-electrode cell. The working and reference electrodes were fixed as close as possible to ensure that the solution resistance was below 0.3 Ω. The wetted area of all electrodes were tailored to be 1 cm^2. The following equation was used to convert the applied potential from *vs* Hg/HgO to *vs* RHE:

$$E_{vs\ RHE} = E_{vs\ Hg/HgO} + 0.059 * pH + 0.098$$

Before tests, we purged the electrolyte with Ar gas for 10 minutes to exclude oxygen. We performed LSV at a scan rate of 5 mV s^{-1} with 85% *iR* correction and cyclic voltammetry (CV) at a scan rate of 50 mV s^{-1}. We applied step chronoamperometry (CP) at current densities of 0, 20, and 1,000 mA cm^{-2} for 10 minutes each in the AST. We also performed electrochemical impedance spectroscopy (EIS) at the potential corresponding to a current density of 10 mA cm^{-2} with frequencies from 1 MHz to 0.1 Hz. EIS spectra were fitted by ZView software to obtain electrochemical parameters. We measured electric double layer capacitance in the non-Faradic region at scan speeds of 20, 40, 60, 80, and 100 mV s^{-1} by CV. Electrochemical surface areas (ECSA) of samples were obtained by dividing the electric double layer capacitance by the specific capacitance of 40 μF cm^{-2}. For performance test of nitrate reduction reaction, we prepared NH_4Cl solutions with different concentrations to prepare a standard curve. The detection reagent consists of three solutions. Liquid A is composed of 1M NaOH, 5wt% salicylic acid and 5wt% sodium citrate. 450 μL sodium hypochlorite standard solution was added to 30 mL deionized water to form solution B. 0.1g sodium nitroprusside was added to 10 mL deionized water to form solution C. Then we mixed 1 mL liquid A, 0.5 mL liquid B, 0.1 mL liquid C and 1mL sample solution followed by using UV-Vis adsorption spectrum to test the absorbance near 650nm.

Scalable preparation of large-area HMH electrodes

First, according to the synthesis protocol, we refined the precursor powder by ball milling and dispersed it into slurry, followed by spraying coating slurry onto metal substrates by an automatic sprayer. Then, as-prepared metal substrates were annealed at high temperature in a tube furnace as per required conditions. After that, we conducted high-throughput screening of HMHs using in-situ polarization imaging method, and selected the best one for AEMWE assembly and testing. Finally, we obtained three HMH electrodes with dimensions of 10×10 cm^2. All electrodes show good uniformity (Fig. S21).

AEMWE assembly and tests

We adopted the PiperION-A40 membrane as anion exchange membrane (AEM), and used the Pt/C catalyst (Johnson Matthey Co., Pt/XC-72R, 40 wt%) with the addition of ionomer (PiperION, A5-HCO3-EtOH, 5 wt%) as cathode catalyst. We also used the Cu foam as porous transport layer in cathode. For performance comparison, we used CoFe-HMH and CoFe-coated catalyst with mass loading of 5 mg cm^{-2} as anode catalysts. Heating and pressing were not required further for the AEMWE assembly. We investigated the AEMWE performance in 1 M KOH at 25 °C and 60 °C using a potentiostat (Zahner XC, Germany). The temperature was regulated using an electrolyte heater. We performed LSV test with a scan rate of 5 mV s^{-1} and EIS test at the potential corresponding to a current density of 50 mA cm^{-2} with frequencies from 1 MHz to 0.1 Hz. We further conducted AEMWE tests under AST by a potentiostat (ITECH, IT6900A, China). In AST protocol, the AEMWE operates at 500 mA cm^{-2} for 15 minutes, followed by operating at 50 mA cm^{-2} for another 15 minutes. It then cycles between 500 and 50 mA cm^{-2} for 15 times, each cycle lasting one minute. Finally, it pauses for 15 minutes at 0 mA cm^{-2}. We collected the electrolyte every 2 hours for ICP-OES during AST. We also conducted conventional CP test for AEMWE in 1 M KOH over 1,000 hours. The decay rate of potential (D_v) was calculated by subtracting the average potential of the last 100 hours from the average potential of the initial 100 hours and dividing by the total time.

In-situ polarization imaging method

System compositions

The test principle is mainly based on the effects of polarized light scattered by tiny particles on the DOP. The process includes bubble nucleation and growth, accompanied by an increase of bubble size, which corresponds to the transition process from Rayleigh scattering to Mie scattering, thereby causing changes in the DOP. The in-situ polarization imaging system consists of a light source module, an electrochemical cell module, and a detection module (Fig. S16). It detects the onset potentials of HER and OER and uses them as criteria to screen the samples. The light source is a light-emitting diode with a wavelength of 525 nm. A polarizer was installed between the slit and the lens to polarize the emitted light. The electrochemical cell module consists of a homemade electrolytic cell, a window glass sheet (50×50×5 mm^3) and a three-electrode cell. The working electrode is a copper plate containing 5×5 grooves filled with 20 HMHs, and the counter electrode and reference electrode are the carbon rod and the Hg/HgO electrodes. The parallel polarized light emitted by the light source passed through the window glass sheet, causing backscattering on the surface of the working electrode, thereby transmitting the information on the surface of working electrode to the detection module. A polarization analyzer was added between the industrial camera and the lens in detection module. The electronic filter wheel (ZWO, EFW-Mini, China) was equipped with two polarizers, of which polarization axes were parallel and perpendicular to the polarizers in light source module. The built-in stepper motor controlled by the ASI driver software can accurately switch between the two polarizers to capture images of electrodes in different polarization states. All acquired images were stored in the computer for subsequent data processing and analysis.

Experimental process

We conducted the electrochemical test in 1 M KOH solution. Before the test, we activated the samples by CV as follows. For HER, the scan voltage was from -0.8 V to -1.3 V *vs* Hg/HgO with a scan rate of 50 mV s^{-1}, and the number of cycles was 30 times. For OER, the scan voltage was from 0.3 V to 0.8 V *vs* Hg/HgO, with a scan rate of 50 mV s^{-1}, and the number of cycles was 30 times. After activation treatment, we applied

LSV test for HMHs as follows. For HER, the voltage range was set from -0.8 V to -1.3 V *vs* Hg/HgO with a scan rate of 1 mV s^{-1}. For OER, the voltage range was set from 0.3 V to 0.8 V *vs* Hg/HgO with a scan rate of 1 mV s^{-1}. An industrial camera continuously captured images of the working electrode surface at a rate of 1 frame per second during LSV test. When taking each photo, the driver would control the 0° and 90° polarizers in the electric filter wheel to switch back and forth, and collected images of parallel polarization and vertical polarization. We defined the DOP as the difference between horizontal and vertical light intensity divided by the sum of between horizontal and vertical light intensity, as shown below:

$$\mathrm{DOP} = \frac{I_{\parallel} - I_{\perp}}{I_{\parallel} + I_{\perp}} \quad (1)$$

Here, $I_{\parallel}$ and $I_{\perp}$ stand for the light intensity in the horizontal and vertical directions. We used the absolute difference in DOP between each frame and the initial DOP as the criterion (|ΔDOP|), which were obtained by subsequent data processing and analyzed by MATLAB. We defined the overpotential corresponding to the point of |ΔDOP| mutation as the onset overpotential (η_o) to reflect intrinsic activity of HMHs (Fig. S37). The onset overpotential across the entire area was obtained by averaging the values of all pixels. The difference values of η_o before and after 1,000 CVs were used to characterize stability.

Computational methods

We performed density functional theory (DFT) calculations with vdW corrections using the Vienna Ab Initio Simulation Package, and applied the Perdew-Burke-Ernzerhof function for electron exchange-correlation within the generalized gradient approximation. The ionic cores were described using the projector-augmented wave method with a plane wave expansion cut-off energy of 400 eV [1]. Ionic relaxations were conducted until all forces on the atoms were less than 0.05 eV $Å^{-1}$ with (1 × 1 × 1) Gamma k-point grid. The electronic structure calculations were based on the Hubbard U model to improve the electronic calculation results [2-4]. The Co-Co_8FeS_8 heterostructure model was the original Co_8FeS_8 (1 1 1) slab with 3 × 3 × 1 Co (1 1 1)

supercell. The Cu-$CuMo_6S_8$ heterostructure model was built by placing a pre-optimized 2 × 2 × 1 $CuMo_6S_8$ (0 1 2) slab on the 5 × 9 × 1 Cu (1 1 1) supercell. The Co-$CoMo_2S_4$ heterostructure model was built by combination of 4 × 3 × 1 $CoMo_2S_4$ (1 1 1) slab and 5 × 4 × 1 Co (1 1 1) supercell. The volume parameters are precisely calculated in balance of mismatch rate and computing cost, and all heterostructure models were lower than a mismatch rate of 3.6 % (Table S7). DFT-D3 force-field correction was applied to accurately describe the vdW interaction. The electrostatic potential and differential charge density diagrams were calculated with dipole correction on *z* axis to improve the electronic results.

Supplementary Figures

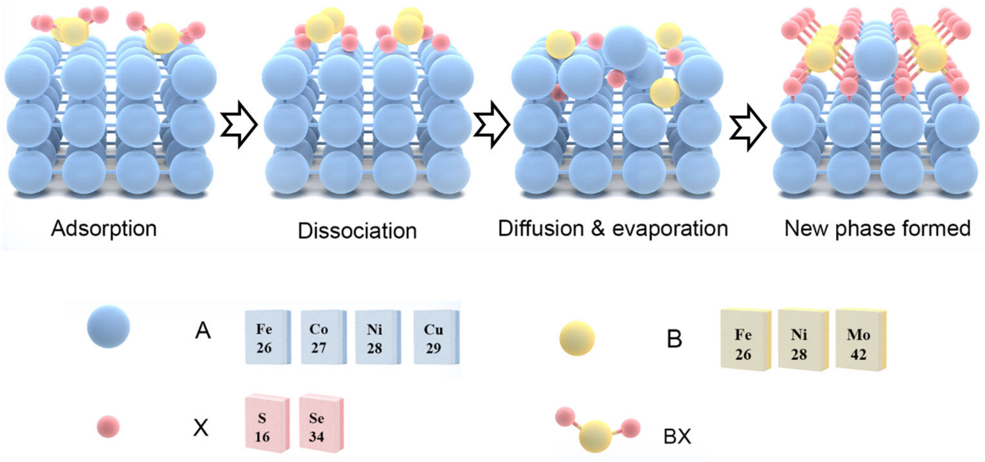

Fig. S1 Schematic of the synthesis of HMHs.

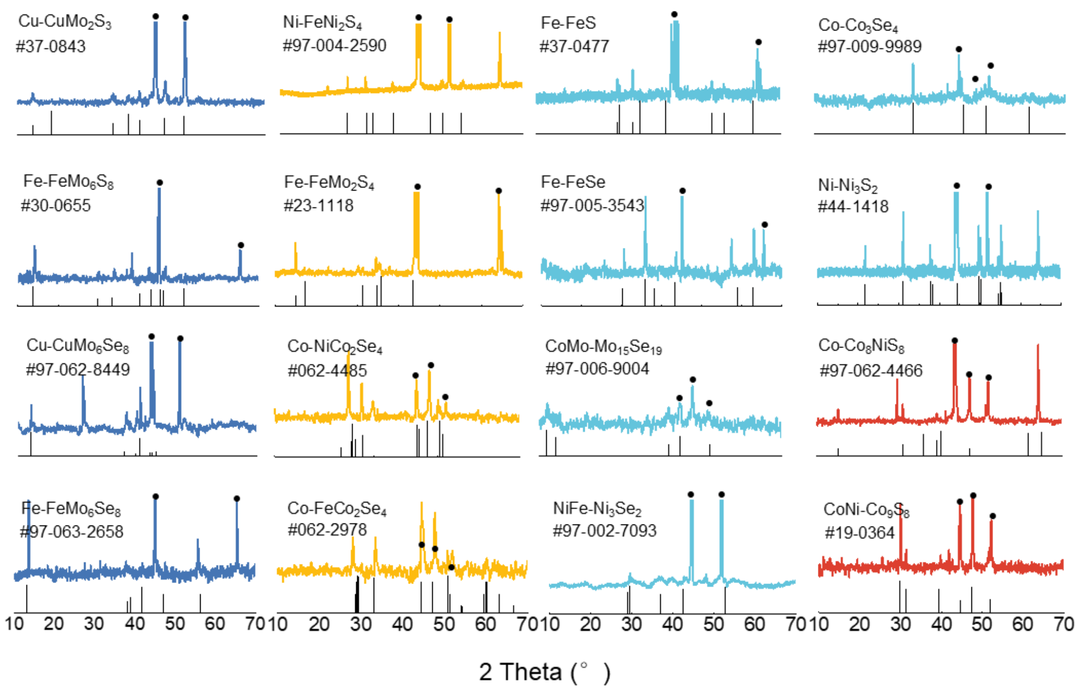

Fig. S2. Crystal structure and phase characterization of different HMHs. XRD patterns of AB_6X_8 (blue), AB_2X_4 (yellow), A-A_8BX_8 (red) and A-A_xB_y (cyan).

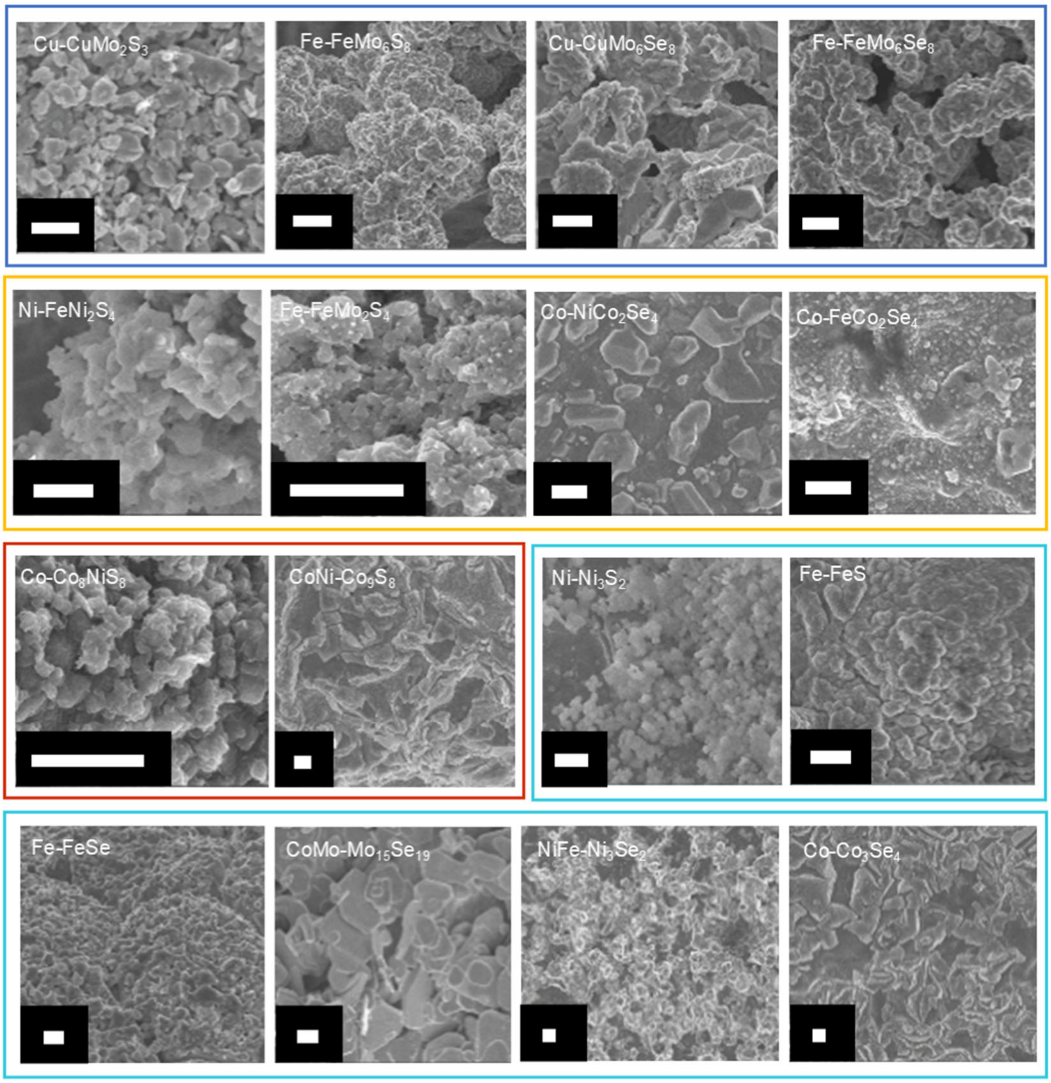

Fig. S3. Morphology characterization of different HMHs. SEM images of AB_6X_8 (blue), AB_2X_4 (yellow), A-A_8BX_8 (red) and A-A_xB_y (cyan).

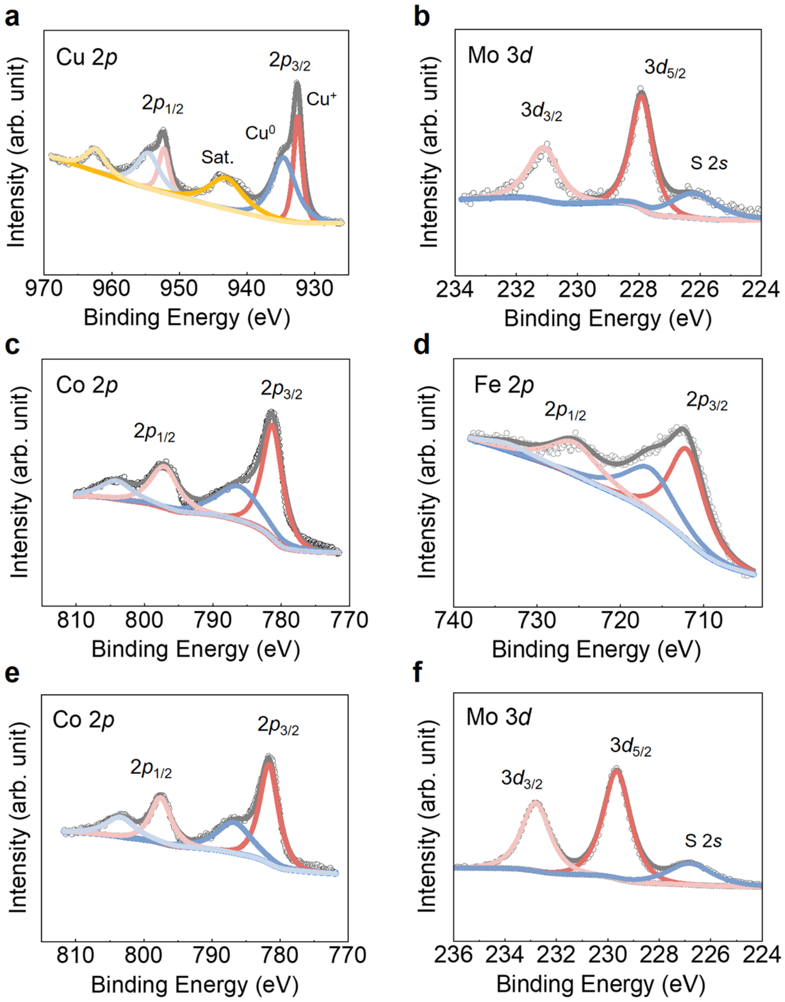

Fig. S4 XPS spectra of HMHs. a Cu 2*p*, **b** Mo 3*d* spectra of $CuMo_6S_8$. **c** Co 2*p*, **d** Fe 2*p* spectra of Co_8FeS_8. **e** Co 2*p*, **f** Mo 3*d* spectra of $CoMo_2S_4$.

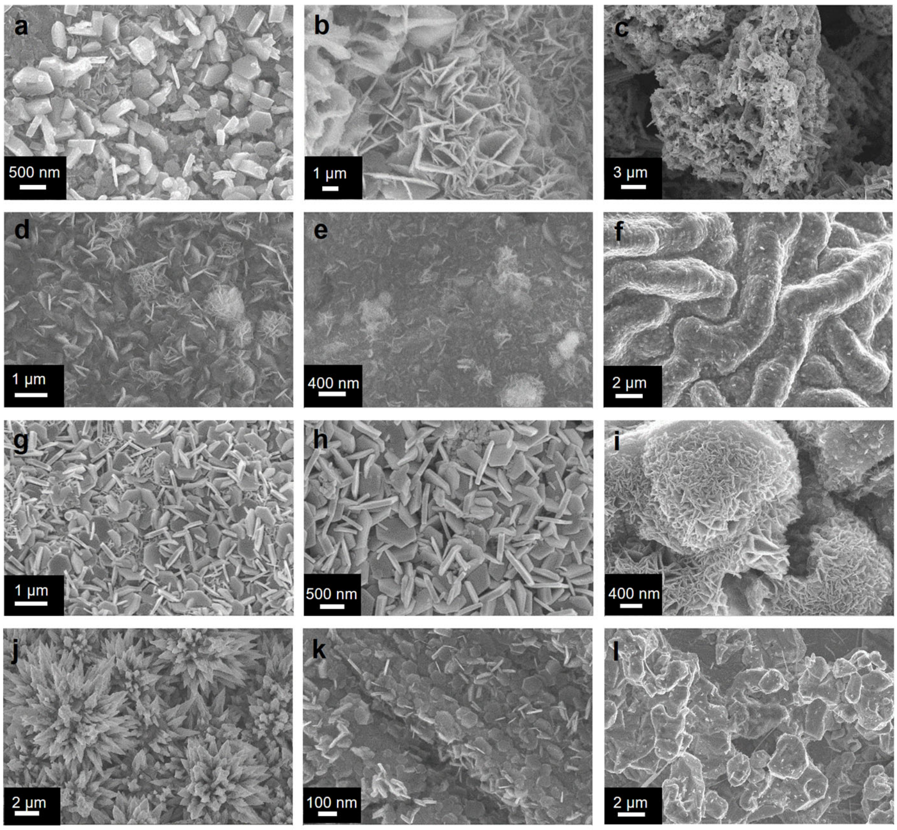

Fig. S5 SEM images of HMHs after activation treatment. a $CoMo_2S_4$, **b** $FeMo_2S_4$, **c** $FeNi_2S_4$, **d** $NiCo_2Se_4$, **e** $CoFe_2Se_4$, **f** Co_9S_8, **g** Co_8FeS_8, **h** Co_8NiS_8, **i** $FeMo_6S_8$, **j** FeS, **k** FeSe, **l** Ni_3Se_2.

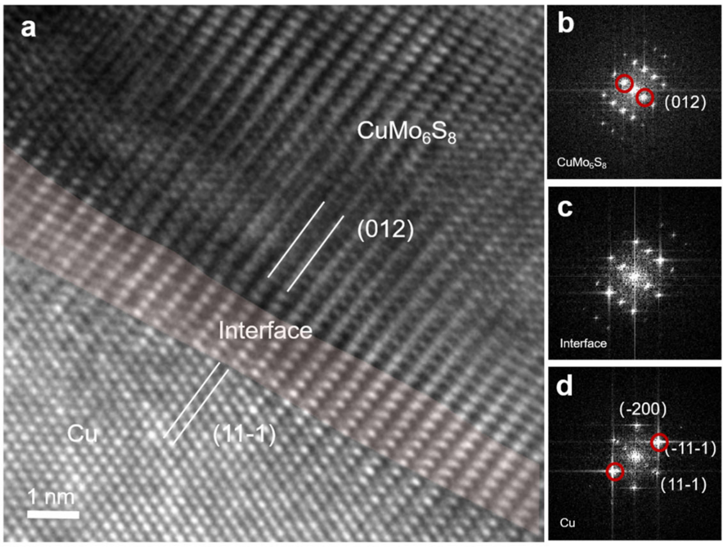

Fig. S6 HRTEM image and corresponding FFT patterns of CuMo-HMH. a HRTEM image of the interfacial structure of CuMo-HMH. FFT patterns of (**b**) $CuMo_6S_8$, (**c**) interface, and (**d**) Cu.

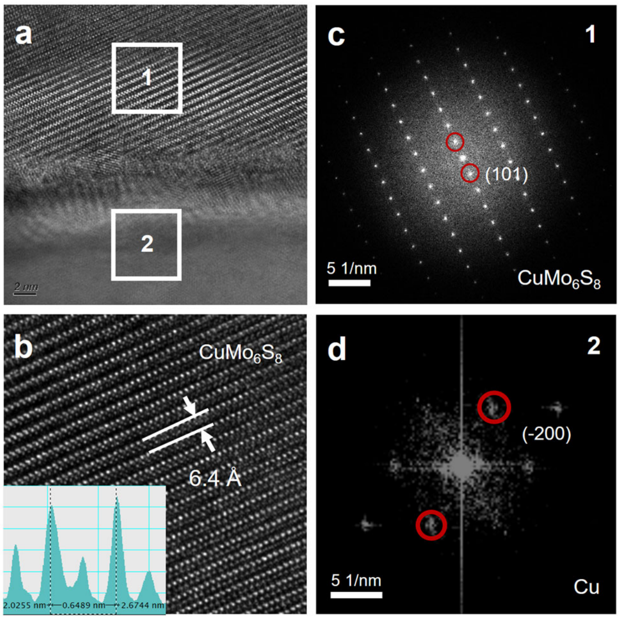

Fig. S7 HRTEM images and corresponding FFT patterns of CuMo-coated sample. **a** HRTEM image of the interfacial structure of CuMo-coated sample. **b** HRTEM image of $CuMo_6S_8$. FFT patterns of (**c**) $CuMo_6S_8$, and (**d**) Cu.

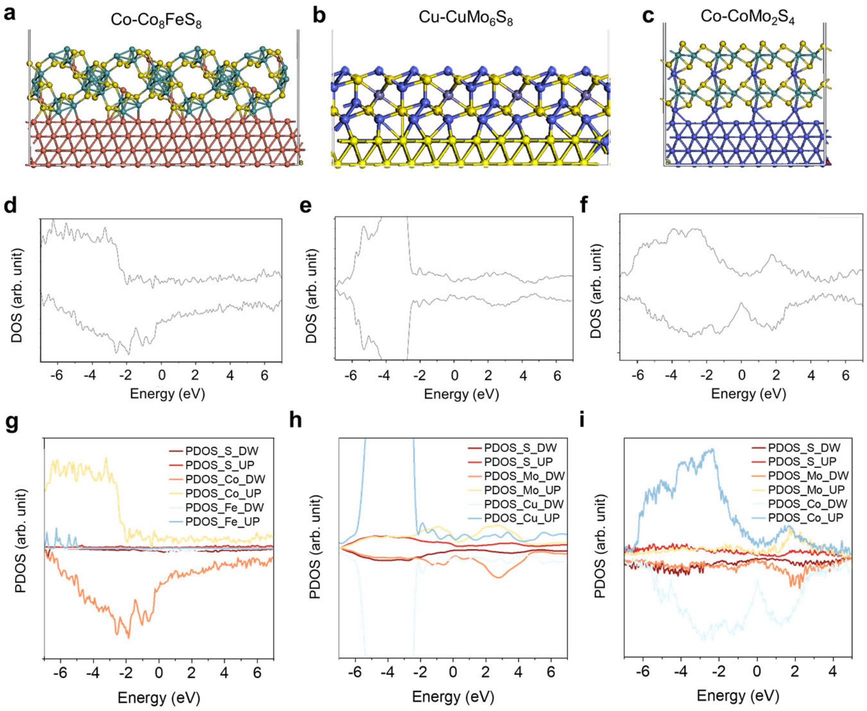

Fig. S8 DFT calculation results of three types of HMHs. a-c Atomic models, **d-f** total density of states (DOS) and **g-i** partial density of states (PDOS) of Co-Co_8FeS_8, Cu-$CuMo_6S_8$ and Co-$CoMo_2S_4$.

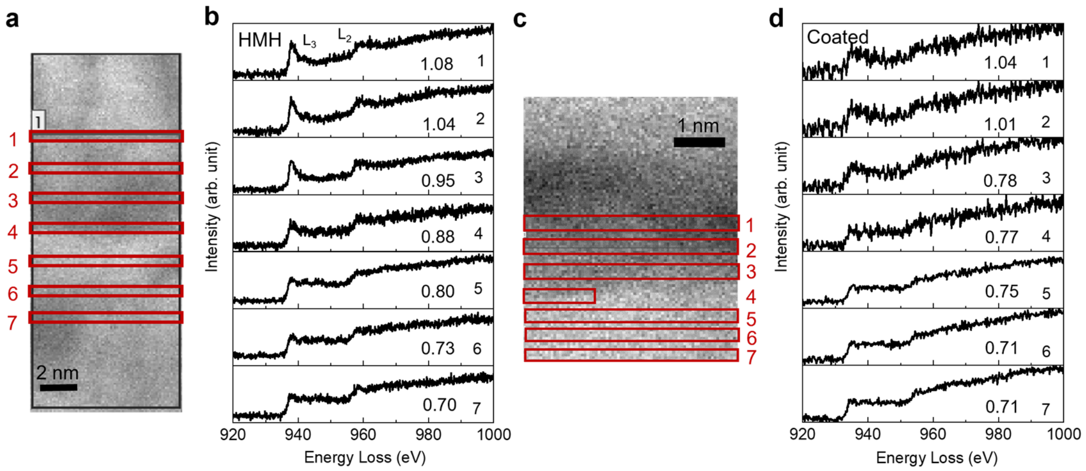

Fig. S9 EELS spectra of Cu *L* edge of CuMo-HMH and CuMo-coated sample. a ADF-STEM image, and (**b**) corresponding EELS spectra of CuMo-HMH. **c** ADF-STEM image, and (**d**) corresponding EELS spectra of CuMo-coated sample.

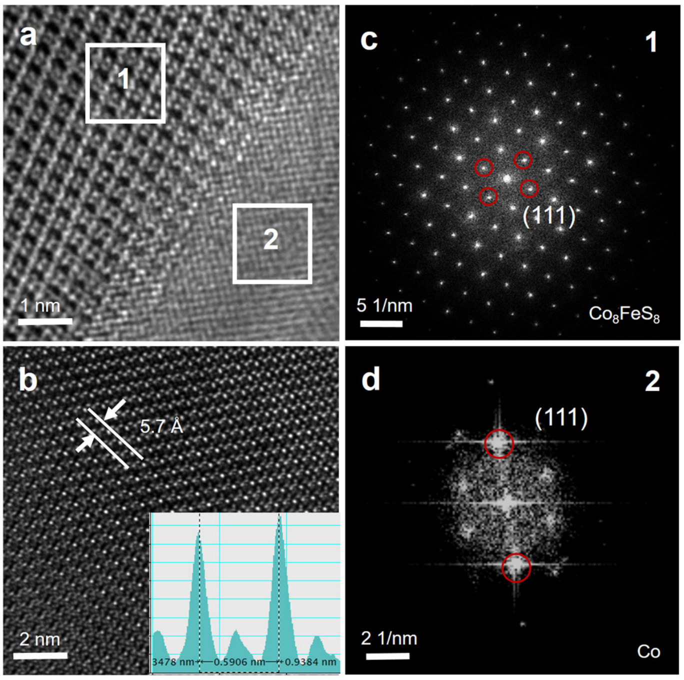

Fig. S10 HRTEM images and corresponding FFT patterns of CoFe-HMH. a HRTEM image of the interfacial structure of CoFe-HMH. **b** HRTEM image of surface catalyst. FFT patterns of (**c**) Co_8FeS_8, and (**d**) Co.

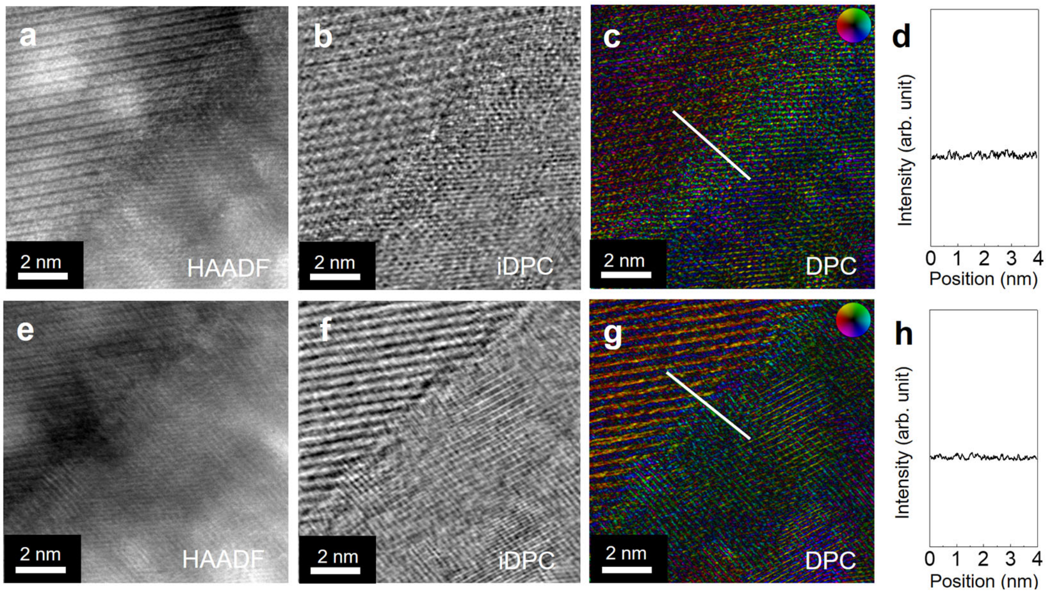

Fig. S11 DPC-STEM images of CoFe-HMH. a, **e** HAADF images, **b**, **f** iDPC images, and (**c**, **g**) DPC-STEM images of interfacial structures located differently in CoFe-HMH. **d, h** The distribution of electric field intensities corresponding to the lines in (**c**) and (**g**).

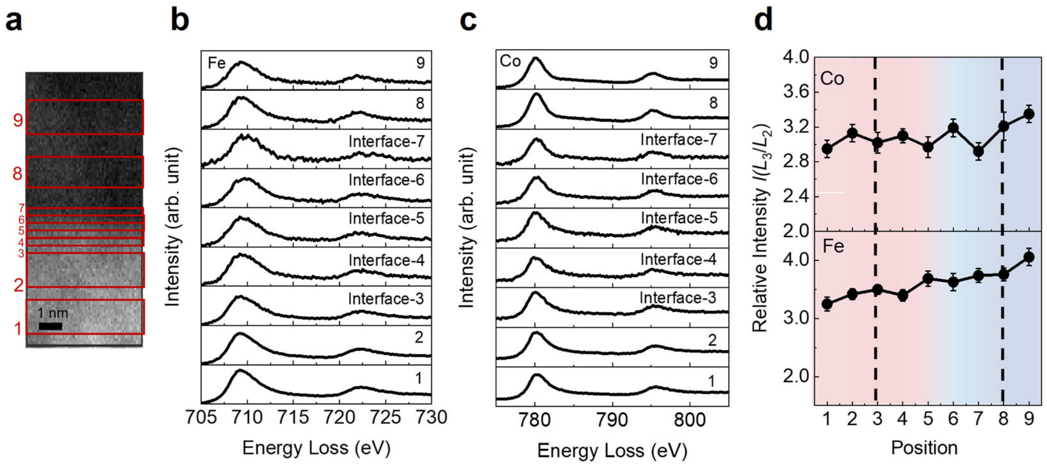

Fig. S12 EELS spectra of Fe and Co *L* edges of CoFe-HMH. a ADF-STEM image, and (**b**, **c**) corresponding EELS spectra of Fe and Co *L* edges. **d** Relative intensity ratio between L_3 and L_2 at different positions of CoFe-HMH.

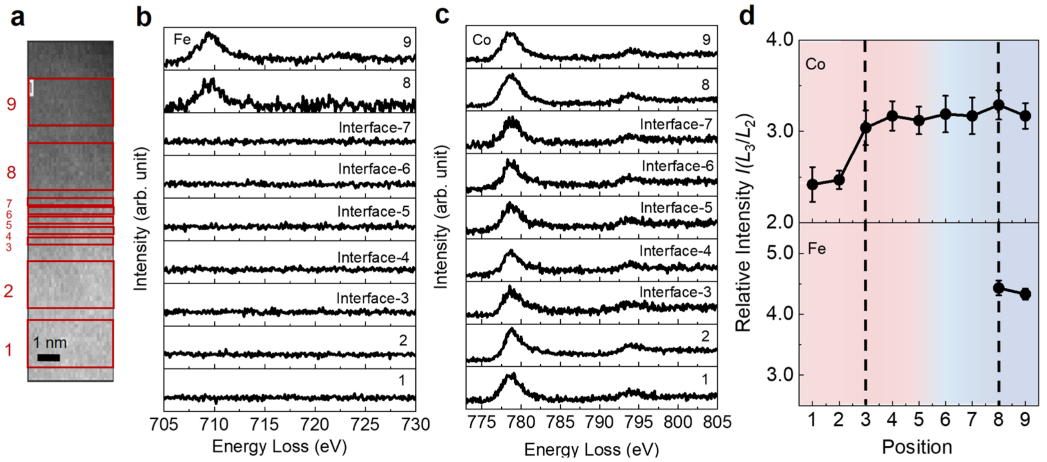

Fig. S13 EELS spectra of Fe and Co *L* edges of CoFe-coated sample. a ADF-STEM image, and (**b**, **c**) corresponding EELS spectra of Fe and Co *L* edges. **d** Relative intensity ratio between L_3 and L_2 at different positions of CoFe-coated sample.

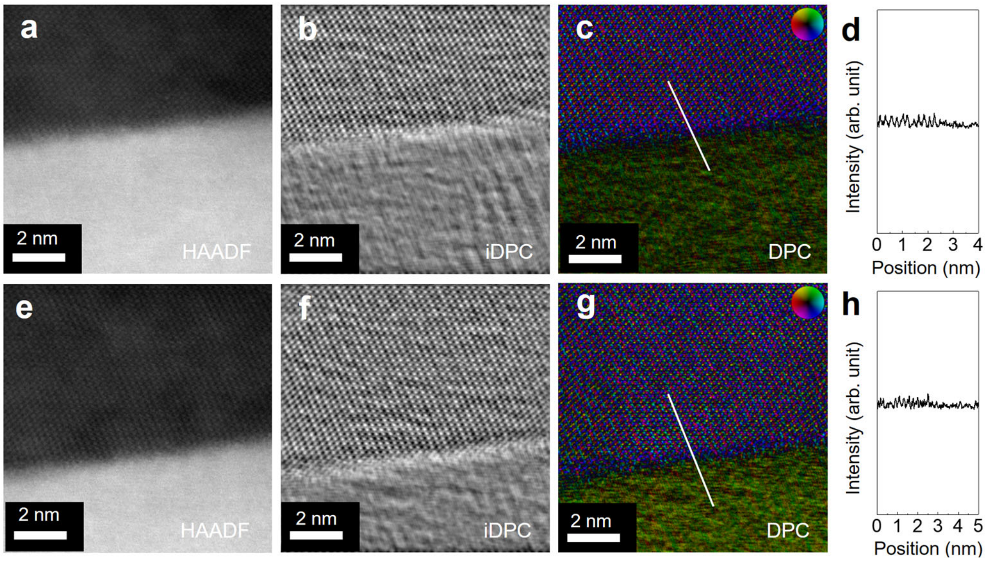

Fig. S14 DPC-STEM images of CoMo-HMH. a, **e** HAADF images, **b**, **f** iDPC images, and (**c**, **g**) DPC based STEM images of interfacial structures located differently in CoMo-HMH. **d**, **h** The distribution of electric field intensities corresponding to the lines in (**c**) and (**g**).

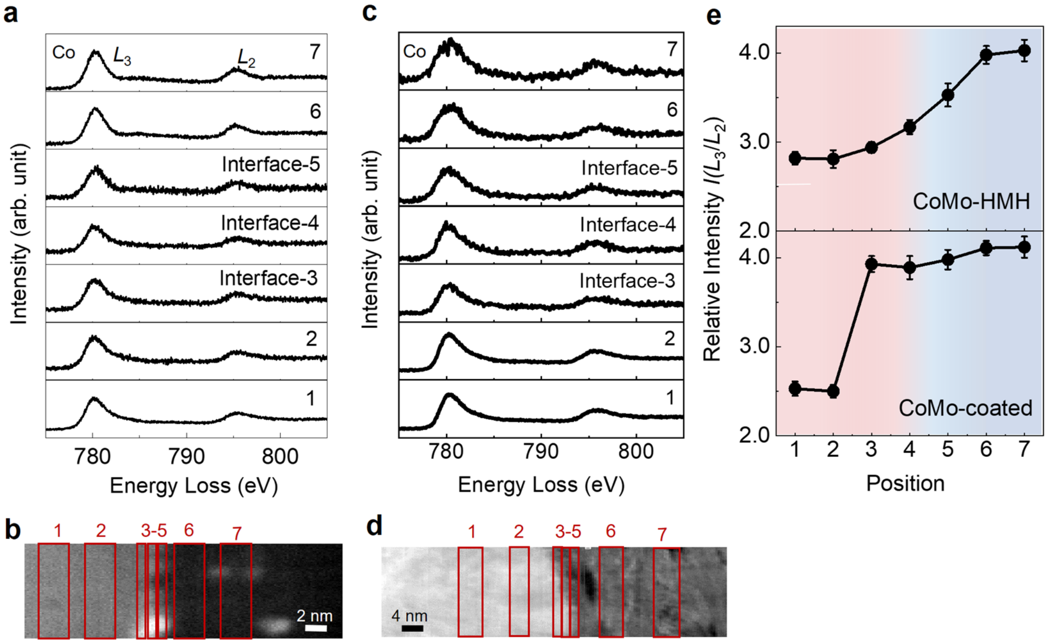

Fig. S15 EELS spectra of Co *L* edges of CoMo-HMH and CoMo-coated sample. EELS spectra of Co *L* edges in CoMo-HMH (**a**) and CoMo-coated sample (**c**), corresponding to ADF-STEM images of (**b**) and (**d**). **e** Relative intensity ratio between L_3 and L_2 at different positions of CoMo-coated sample.

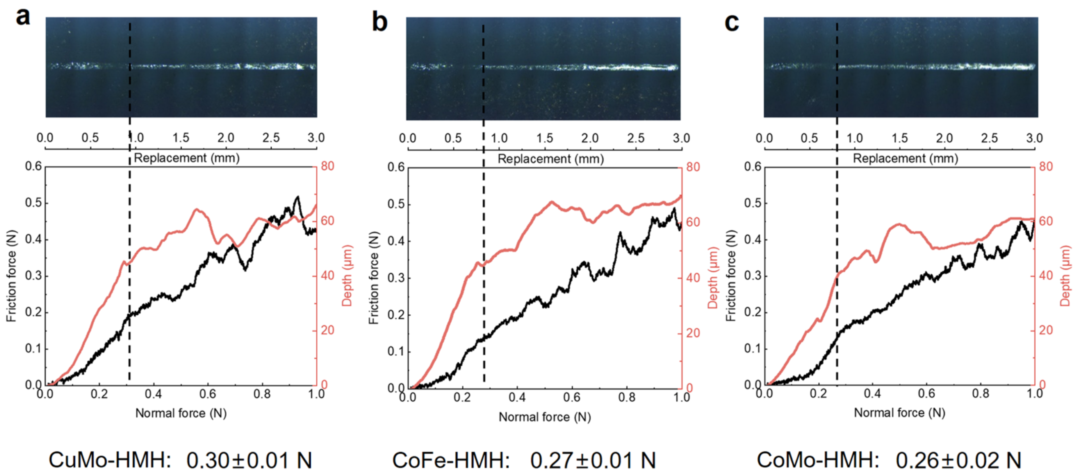

Fig. S16 Micro-scratch mechanical experiments of HMHs. Critical adhesion forces of (**a**) CuMo-HMH, (**b**) CoFe-HMH, and (**c**) CoMo-HMH.

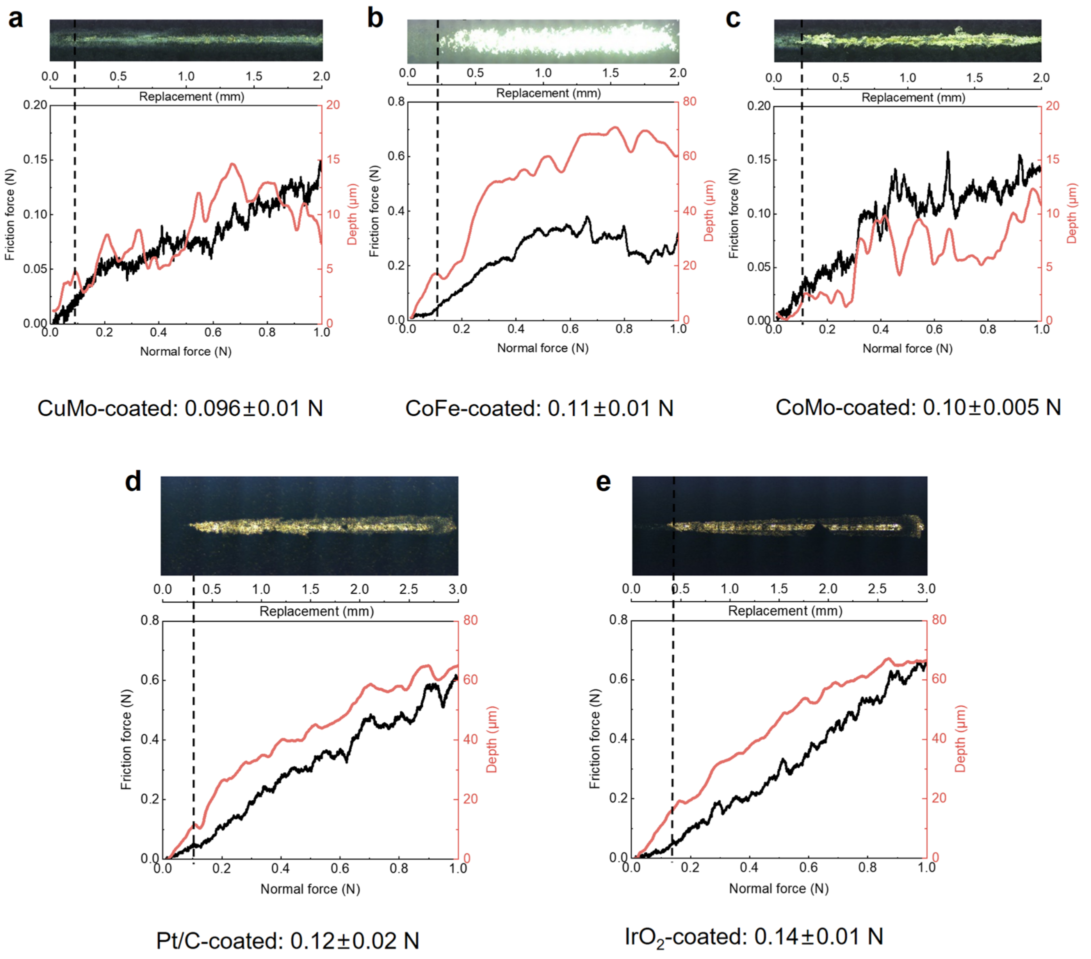

Fig. S17 Micro-scratch mechanical experiments of coated samples. Critical adhesion forces of (**a**) CuMo-coated, (**b**) CoFe-coated, (**c**) CoMo-coated, (**d**) Pt/C-coated and (**e**) IrO_2-coated catalysts.

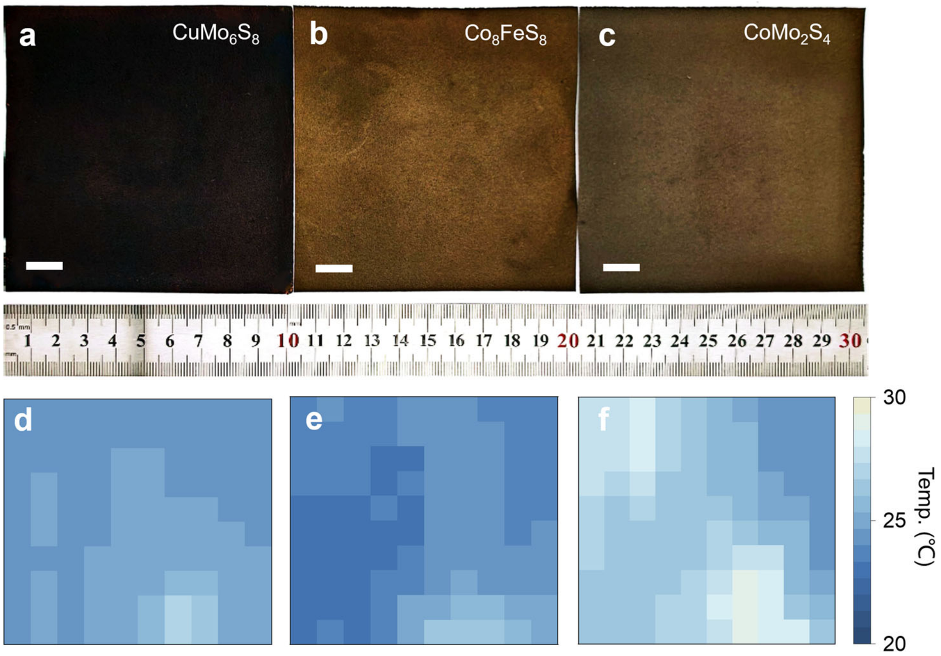

Fig. S18 Large-area HMH electrodes with dimensions of 10 × 10 cm². Real pictures of **(a)** Cu-$CuMo_6S_8$, **(b)** Co-Co_8FeS_8, **(c)** Co-$CoMo_2S_4$, and **(d-f)** corresponding surface temperature distributions when charged at 100 mA cm^{-2}.

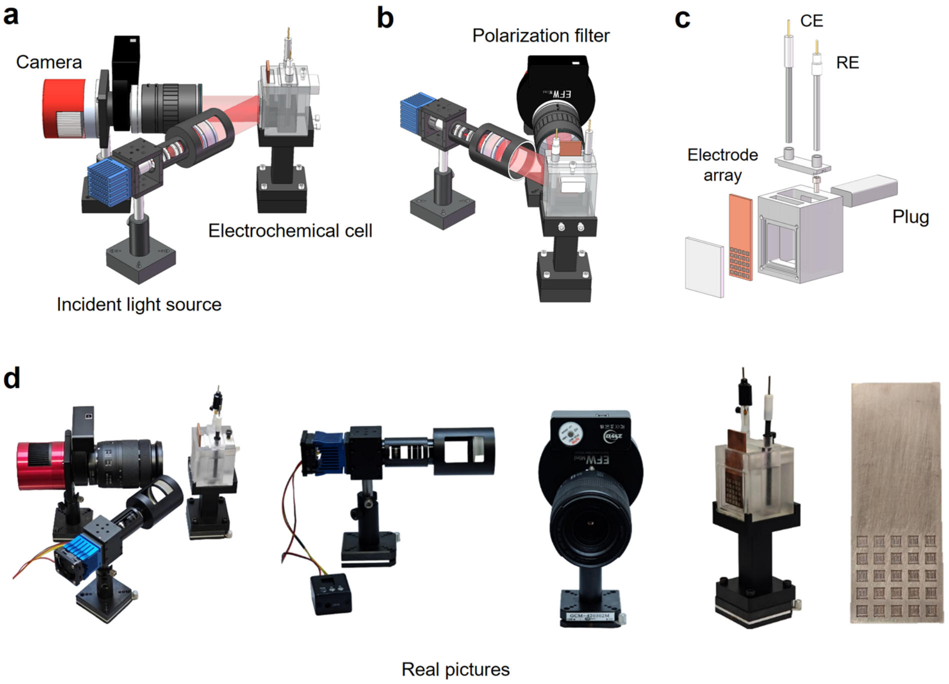

Fig. S19 Schematics of in-situ polarization imaging setups. Front (**a**) and side (**b**) views of setups. **c** Electrochemical cell. **d** Real pictures of setups.

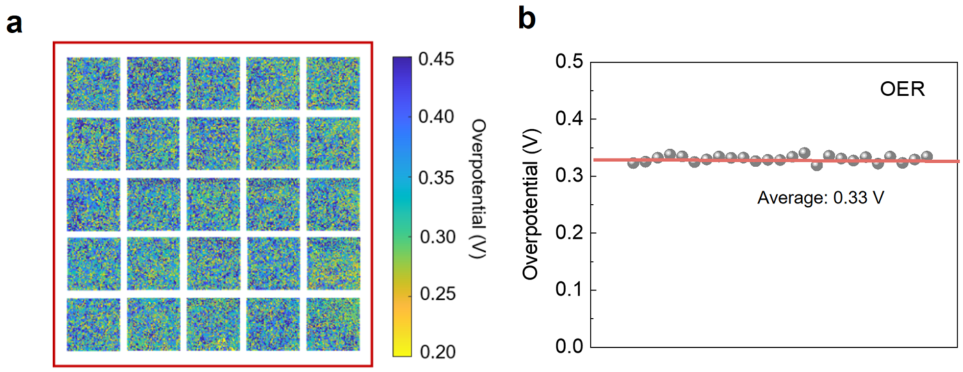

Fig. S20 Consistency experimental results of Ni foams. a Onset overpotential mappings of 25 identical Ni foams. **b** Statistical distribution of overpotential values of OER.

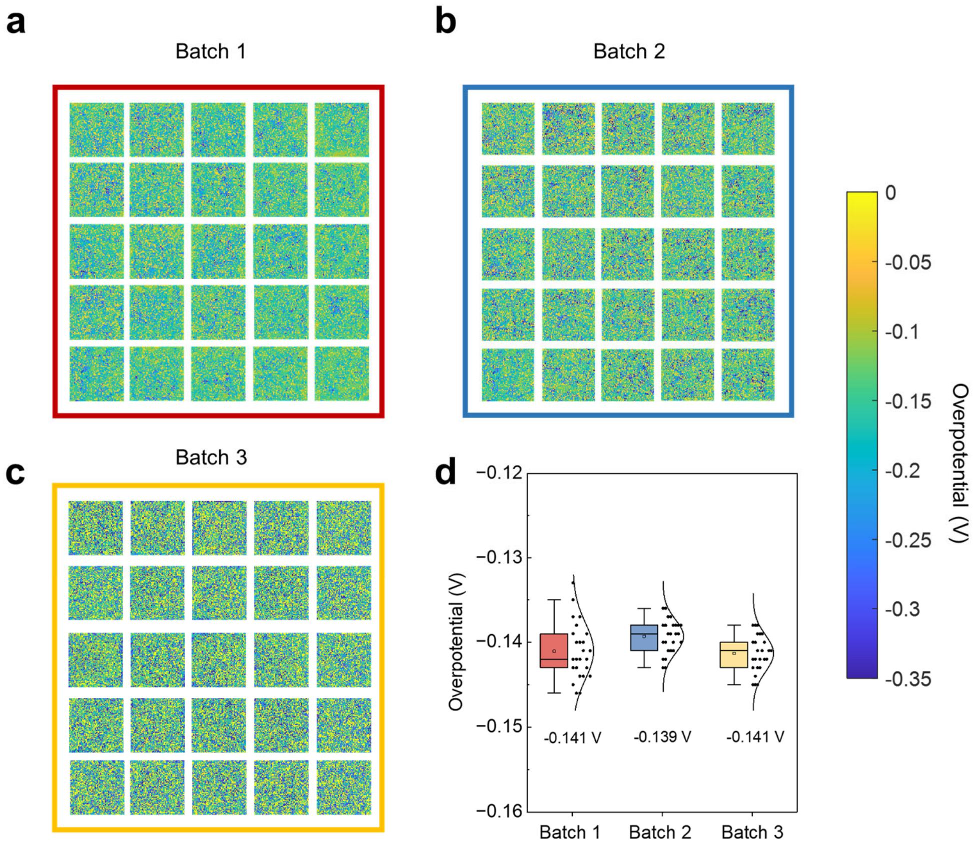

Fig. R21 Reproducibility and error analysis of multiple batches. a-c Onset overpotential mappings of three batches Ni foams for hydrogen evolution. In each batch, 25 locations were studied. **d** Statistical distributions of hydrogen evolution overpotentials in three batch tests.

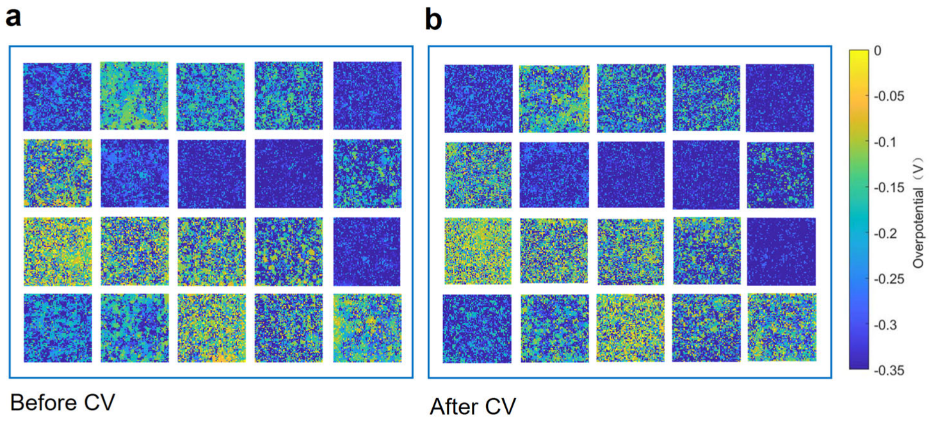

Fig. S22 Mappings of onset overpotentials of 20 HMHs for HER. Mappings before (**a**) and after 1,000 CV (**b**).

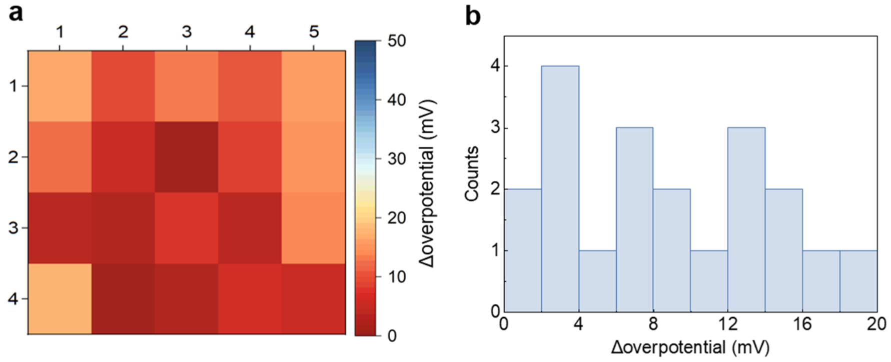

Fig. S23 Differential mappings of HER overpotentials of 20 HMHs before and after CV. Mappings (**a**) and statistical distribution (**b**) of differential values.

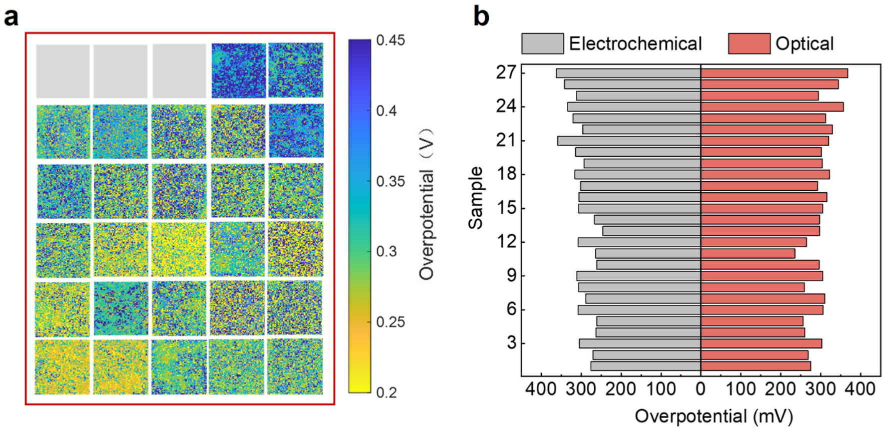

Fig. S24 Optical screening results of 27 synthesis conditions of CoFe-HMH. a Mappings of onset overpotentials of OER under 27 synthesis conditions. **b** Comparison

between electrochemical test results and optical screening results.

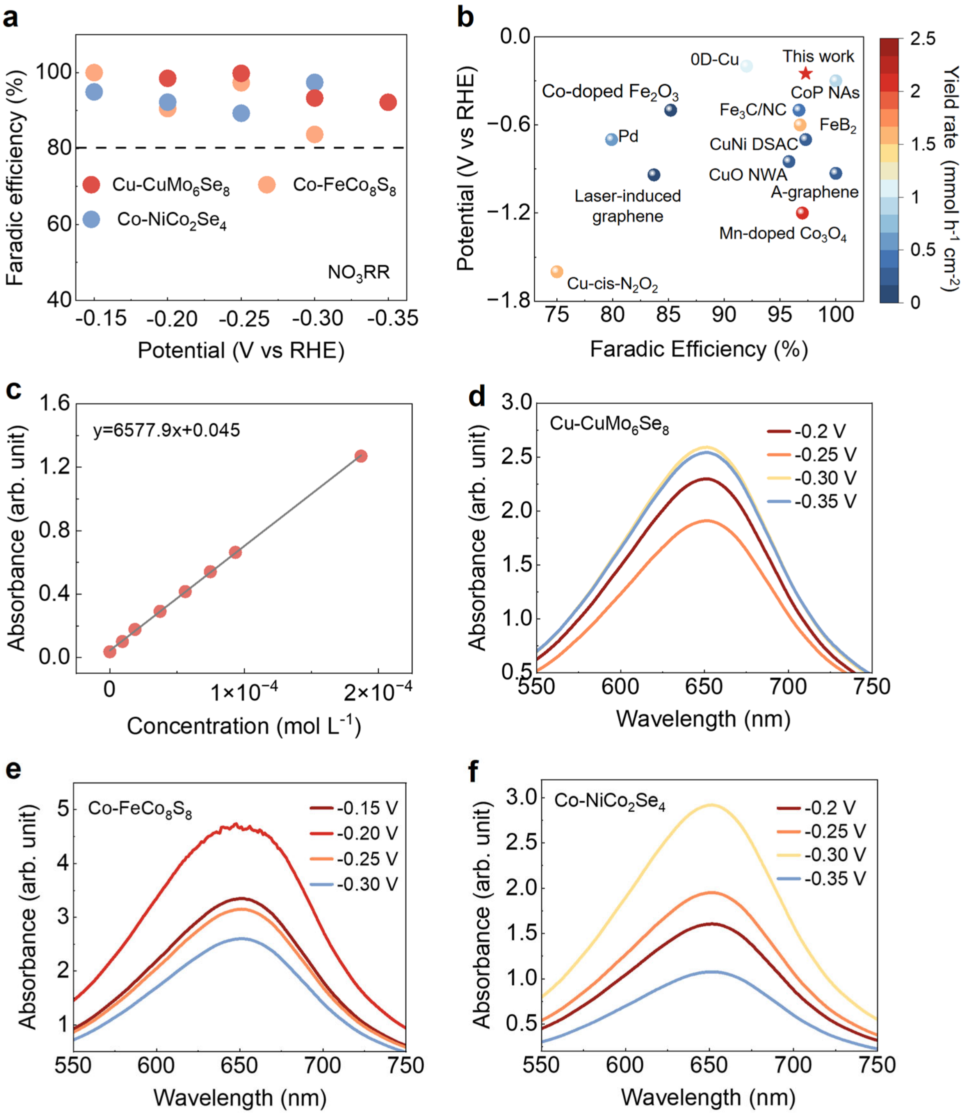

Fig. S25 Performance of three types of HMHs for nitrate reduction reaction. a Faradic efficiency (FE) of three catalysts under different potentials. **b** Comparison of applied potential, FE, and yield rate between this work and other state-of-the-art catalysts. **c** The linear relationship between absorbance of ultraviolet-visible light (UV-Vis) absorption spectrum and standard sample concentration. **d-f** The absorbance of UV-Vis absorption spectrum of three catalysts under different potentials.

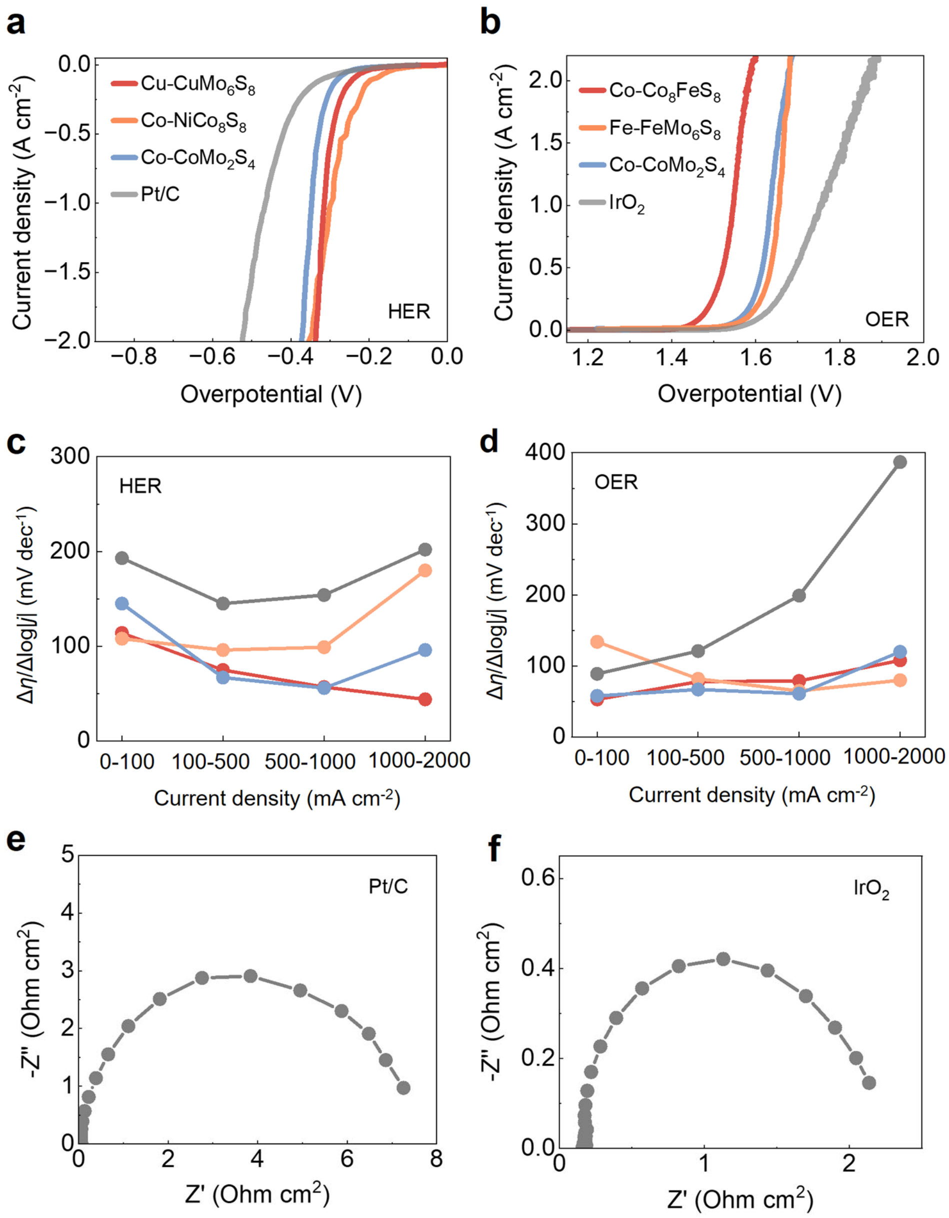

Fig. S26 Electrochemical test results of HMHs and commercial catalysts. a LSV curves of HER. **b** LSV curves of OER. **c, d** The $\Delta\eta/\Delta\log|j|$ values in different current density ranges. EIS spectra of (**e**) Pt/C and (**f**) IrO_2 catalysts.

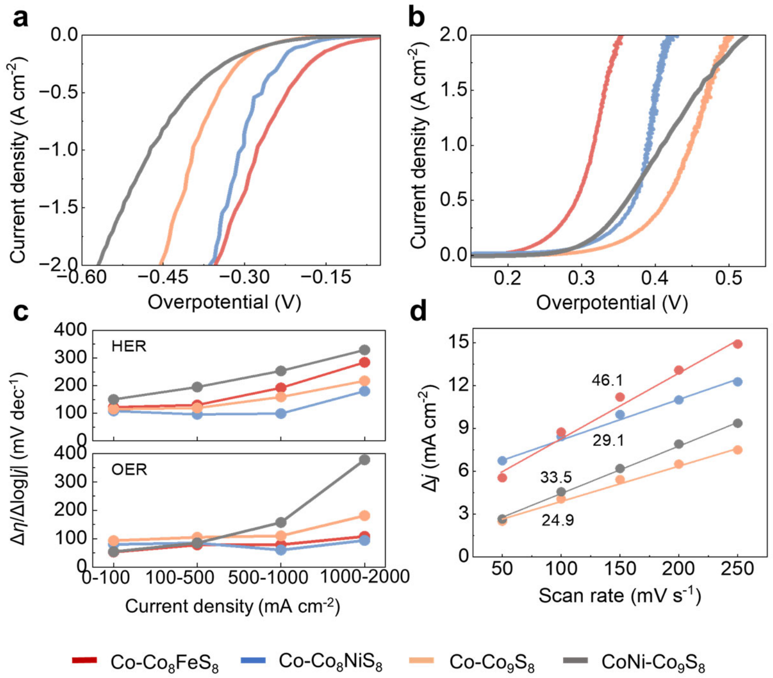

Fig. S27 Electrochemical test results of A-A8BX8. a, b LSV curves of HER and OER. **c** The $\Delta\eta/\Delta\log|j|$ values in different current density ranges. **d** Electrochemical surface area of A-A8BX8 catalysts. The unit of slope is mF cm^{-2}.

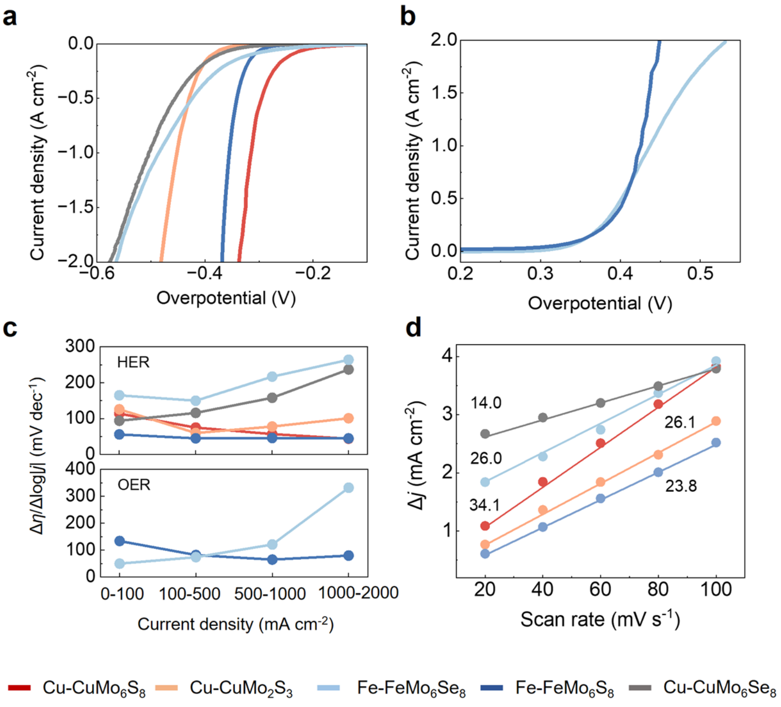

Fig. S28 Electrochemical test results of A-A8BX8. a, b LSV curves of HER and OER. **c** The $\Delta\eta/\Delta\log|j|$ values in different current density ranges. **d** Electrochemical surface area of A-AB6X8 catalysts. The unit of slope is mF cm^{-2}.

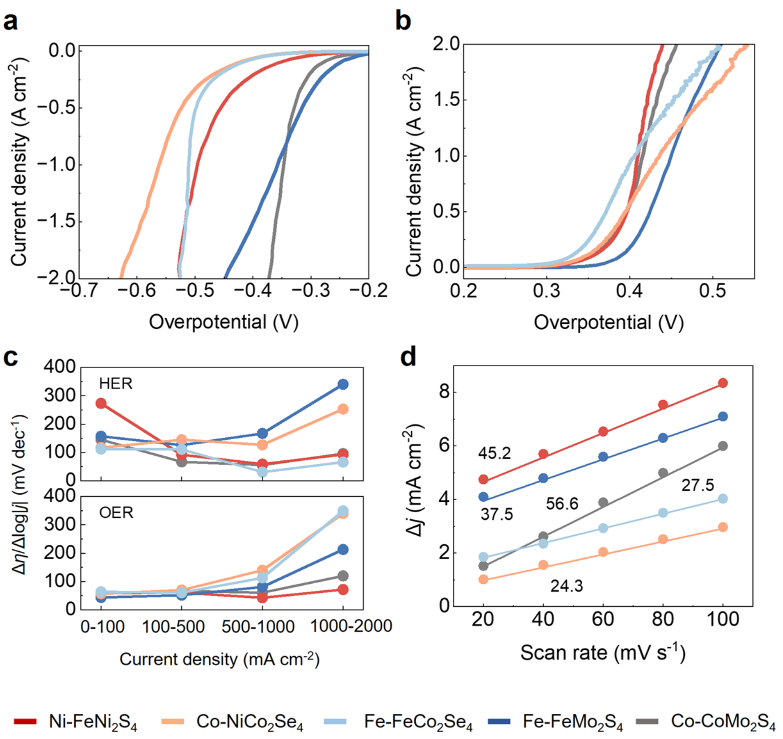

Fig. S29 Electrochemical test results of A-AB$_2$X$_4$. a, b LSV curves of HER and OER. **c** The Δη/Δlog|j| values in different current density ranges. **d** Electrochemical surface area of A-AB$_2$X$_4$ catalysts. The unit of slope is mF cm^{-2}.

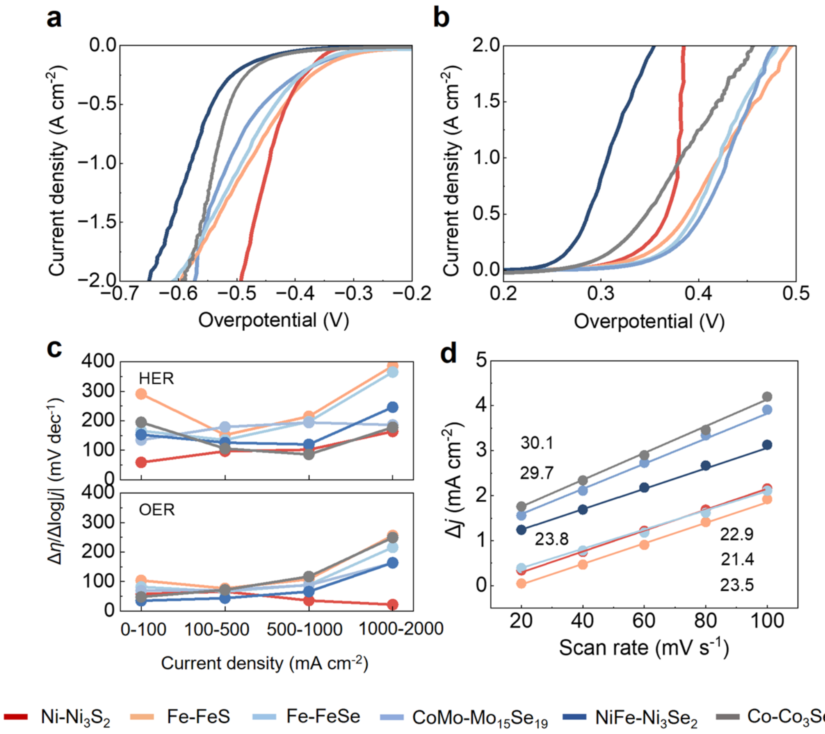

Fig. S30 Electrochemical test results of A-A$_x$X$_y$. a, b LSV curves of HER and OER. **c** The Δη/Δlog|j| values in different current density ranges. **d** Electrochemical surface area of A-A$_x$X$_y$ catalysts. The unit of slope is mF cm^{-2}.

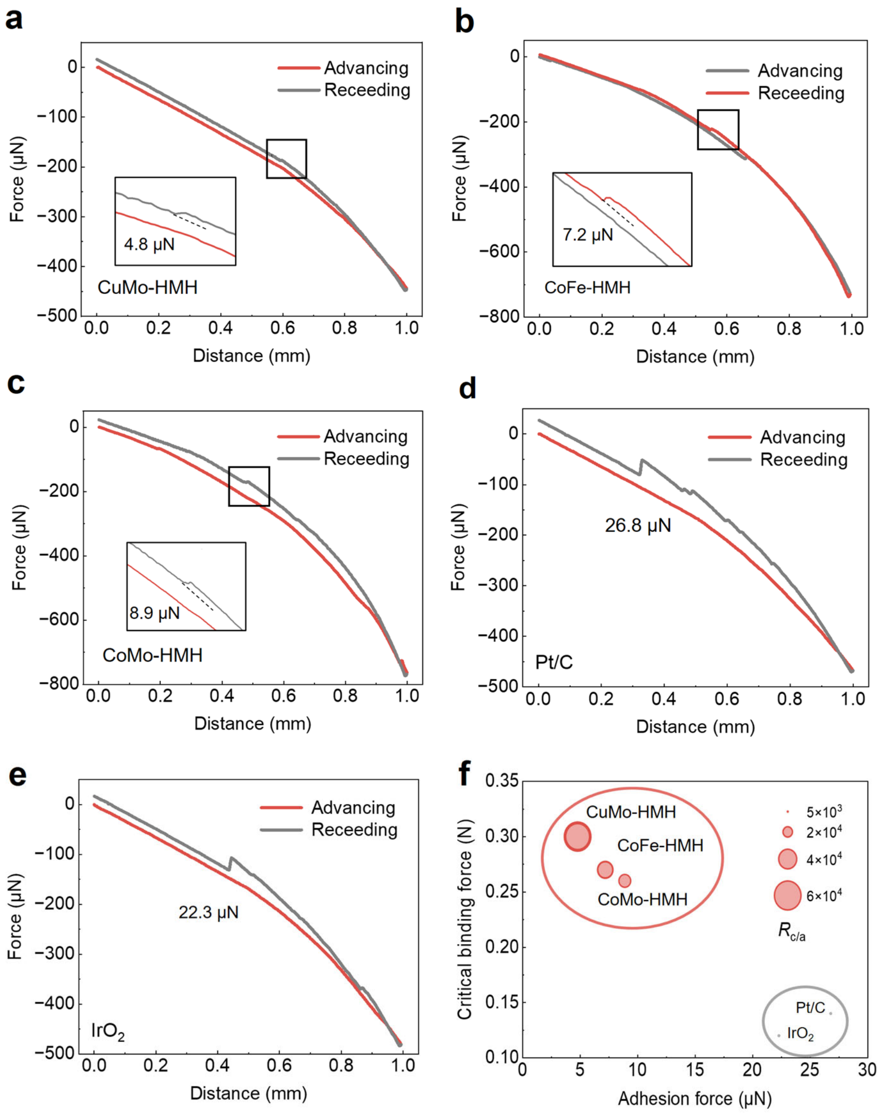

Fig. S31 Bubble adhesion force results of HMHs and commercial catalysts. Force-displacement curves of (**a**) CuMo-HMH, (**b**) CoFe-HMH, (**c**) CoMo-HMH, (**d**) Pt/C, and (**e**) IrO_2 catalysts. **f** Critical binding forces, bubble adhesion forces, and relative ratio between both ($R_{c/a}$). The larger the $R_{c/a}$, the higher the interface stability.

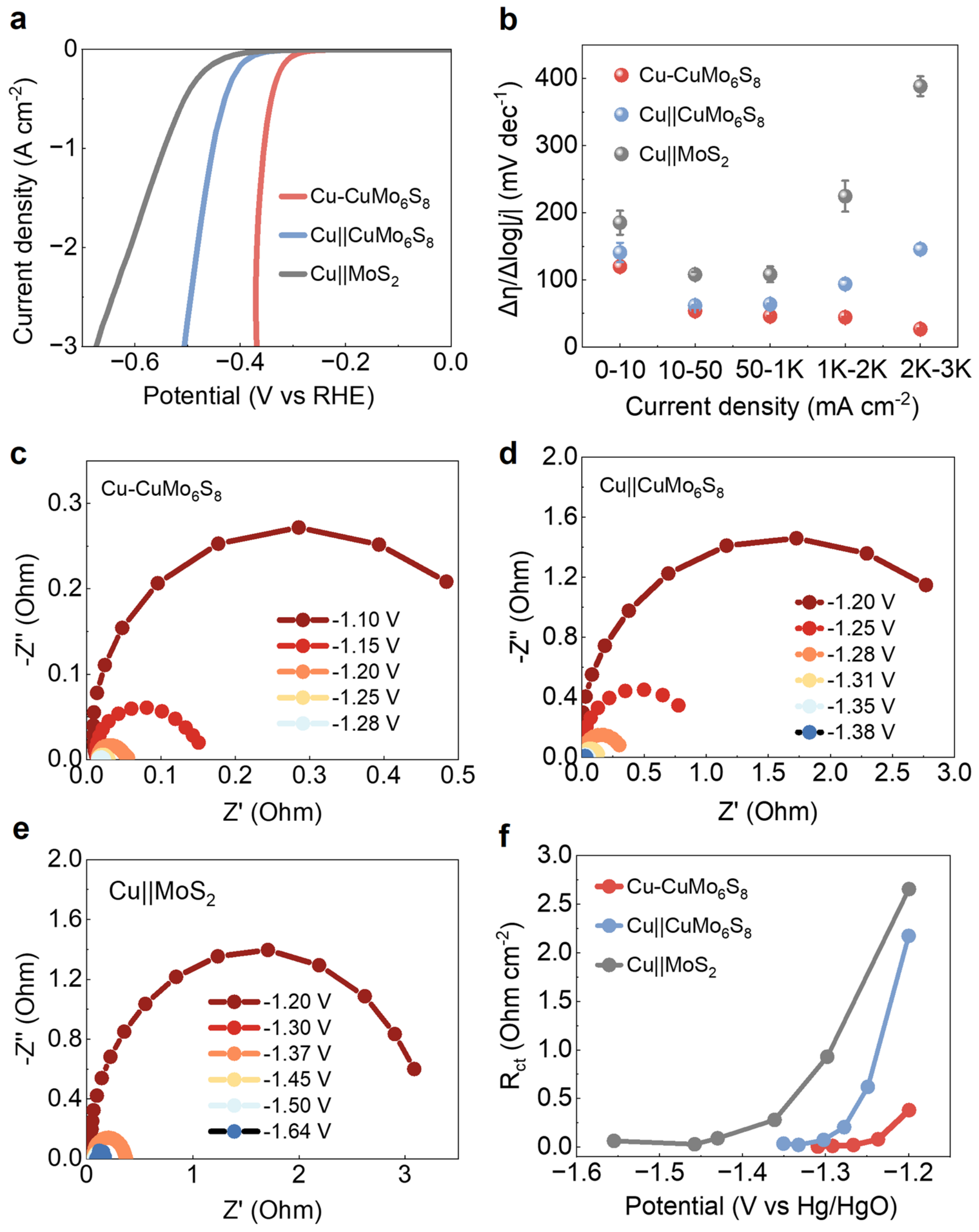

Fig. S32 Electrochemical test results of CuMo-HMH, CuMo-coated and MoS_2-coated catalysts. a LSV curves of HER. **b** $\Delta\eta/\Delta\log|j|$ values in different current density ranges. EIS spectra of (**c**) CuMo-HMH (Cu-$CuMo_6S_8$), (**d**) CuMo-coated catalyst (Cu||$CuMo_6S_8$), and (**e**) MoS_2-coated (Cu||MoS_2) catalyst under different applied bias. **f** Charge transfer resistances (R_{ct}) of three electrodes under different applied bias.

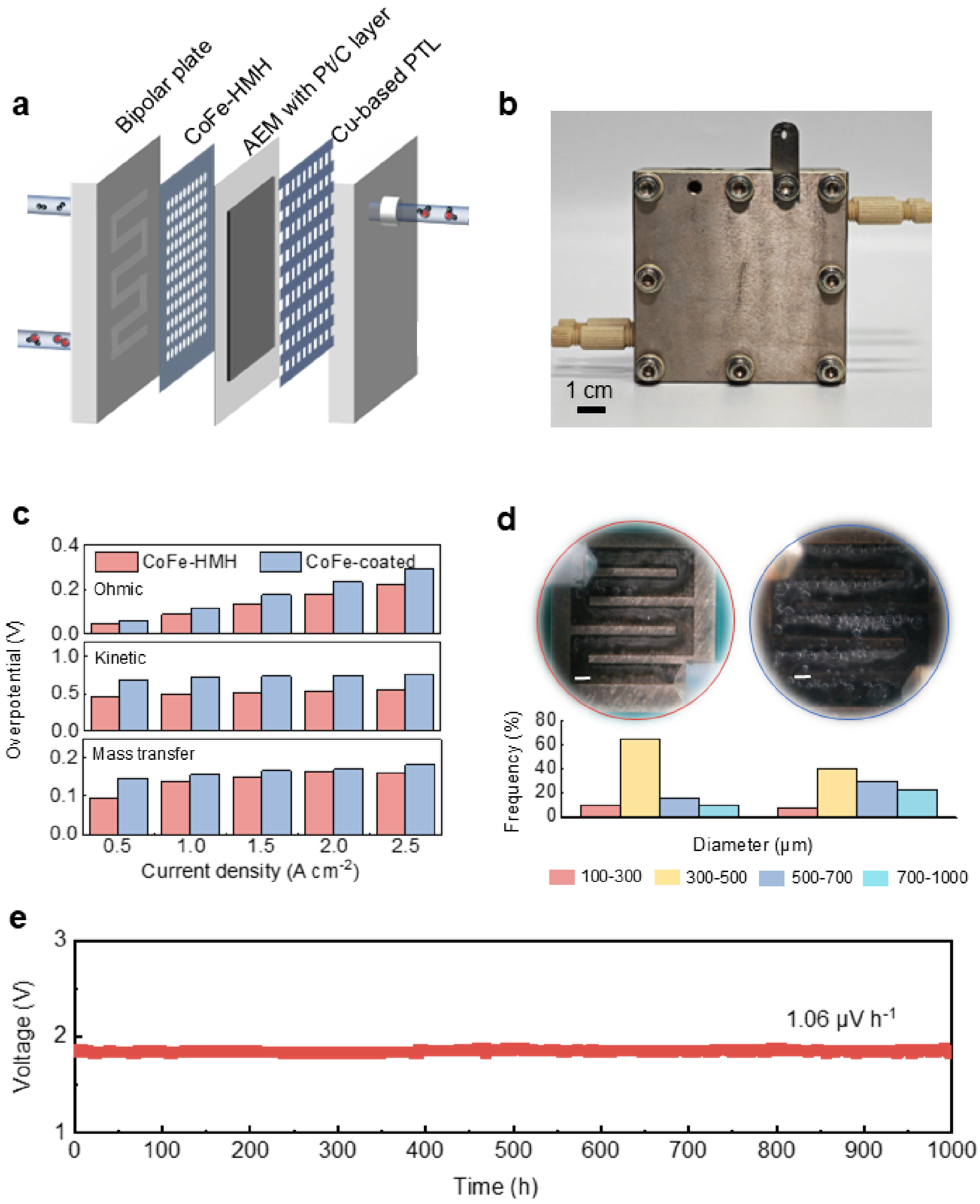

Fig. S33 HMHs based AEMWE. a Schematic and (**b**) real picture of HMHs based AEMWE. **c** Proportion of ohmic, kinetics and mass transfer overpotentials of CoFe-HMH and CoFe-coated catalyst at different current densities. **d** Statistics of bubble size of CoFe-HMH (left) and CoFe-coated catalyst (right) at 500 mA cm^{-2}. **e** CP test results.

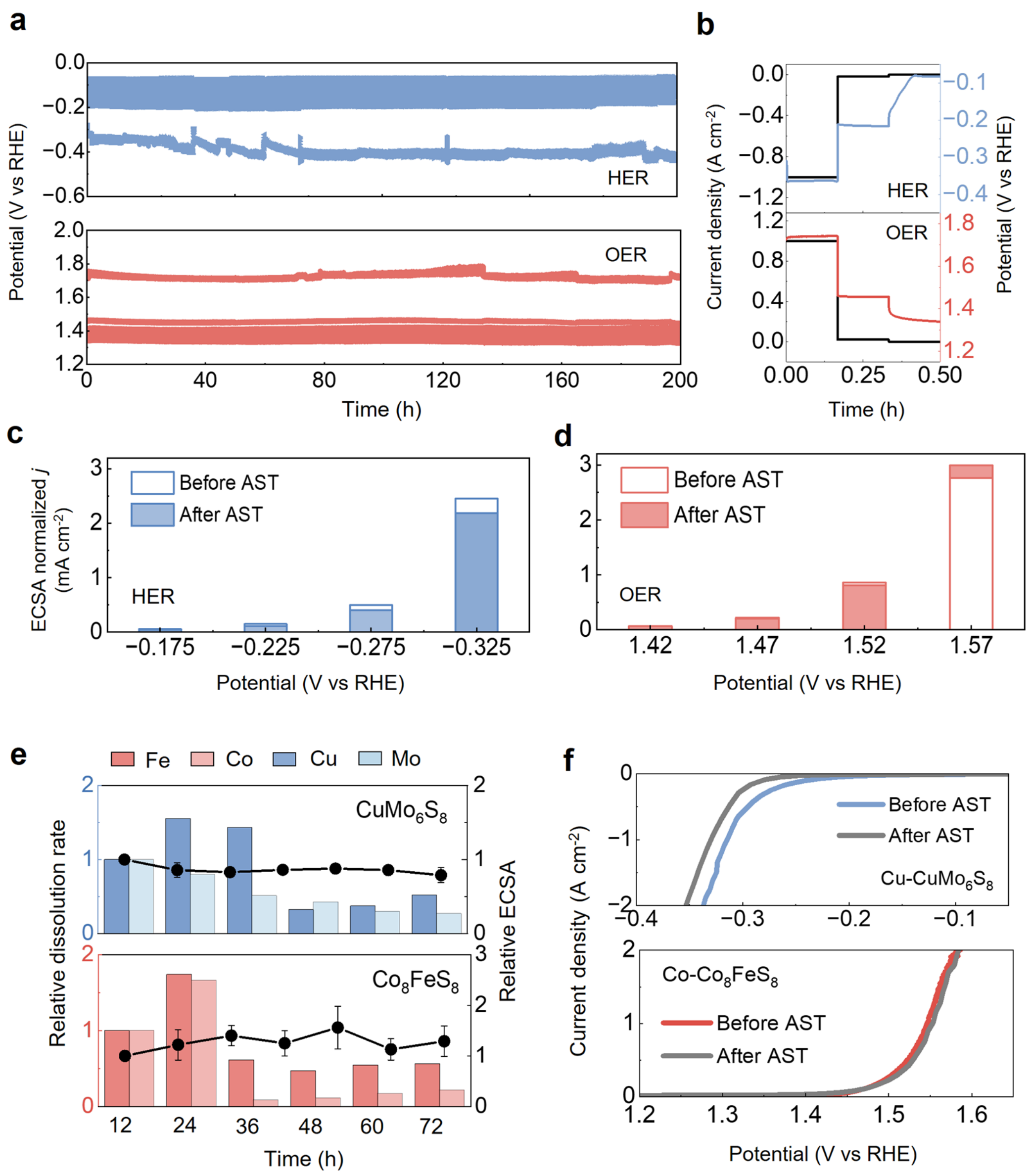

Fig. S34 AST results of HMHs in three-electrode system. a Step CP of Cu-$CuMo_6S_8$ and Co-Co_8FeS_8 for HER and OER. **b** Step CP includes three stages of 1, 0.02, and 0 A cm^{-2}, each running for ten minutes. **c, d** ECSA normalized current densities of HMHs before and after AST. **e** ICP-OES results of metal dissolution from HMHs during AST. **f** LSV curves of HMHs before and after AST.

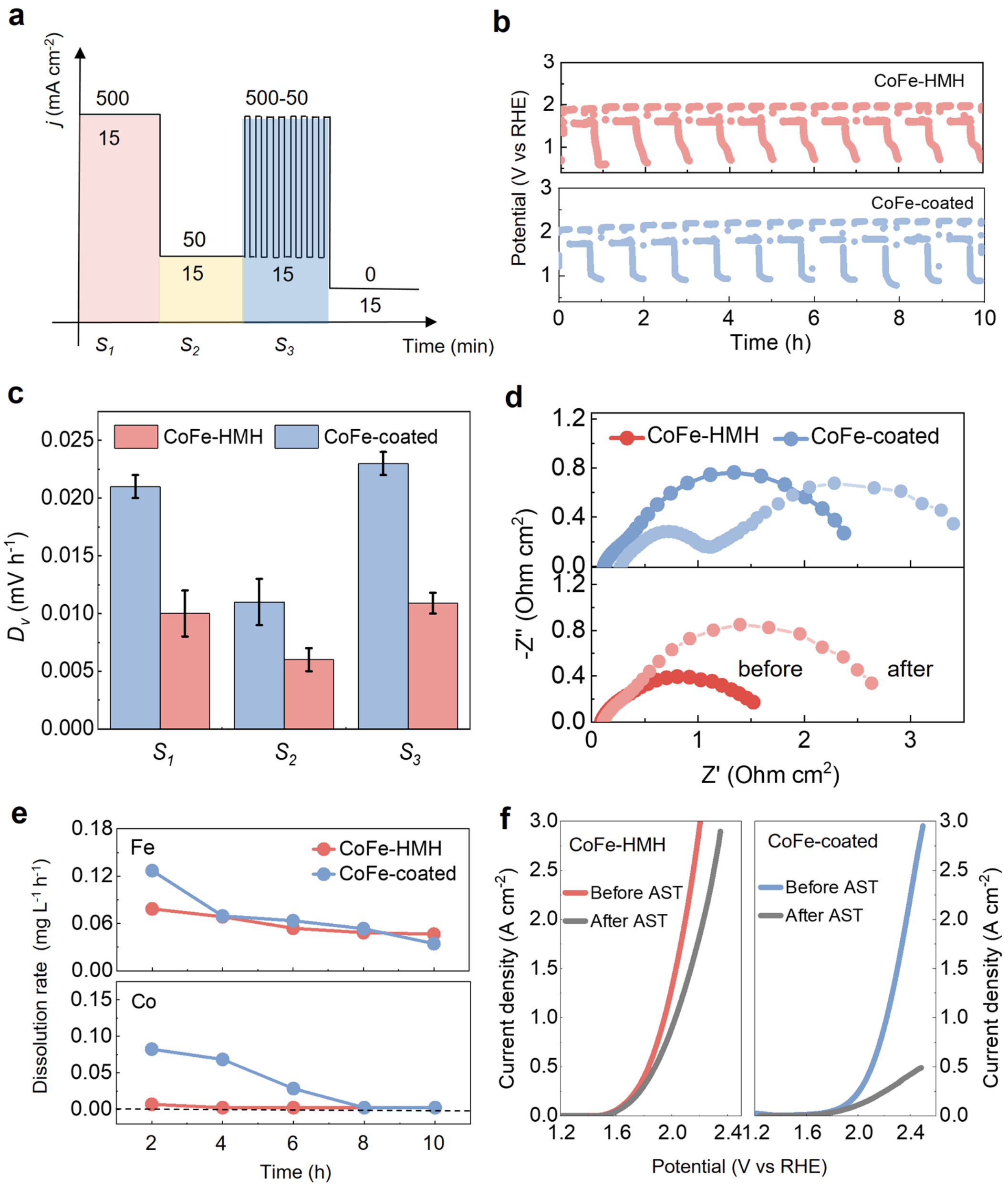

Fig. S35 Electrochemical test results of HMHs and coated catalysts in AEMWE. a AST protocol of AEMWE. **b** AST results, **c** D_v, **d** EIS spectra, and (**e**) ICP-OES results of CoFe-HMHs and CoFe-coated catalyst based AEMWE. **f** LSV curves of CoFe-HMHs and CoFe-coated catalyst based AEMWE before and after AST.

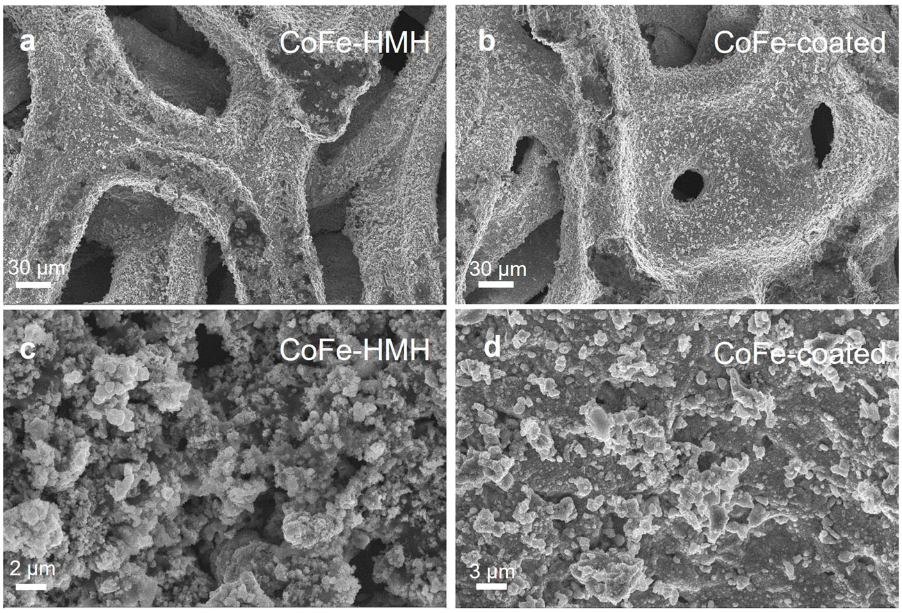

Fig. S36 SEM images of HMH and coated catalyst electrodes in AEMWE after AST. a, c CoFe-HMH. **b, d** CoFe-coated catalyst.

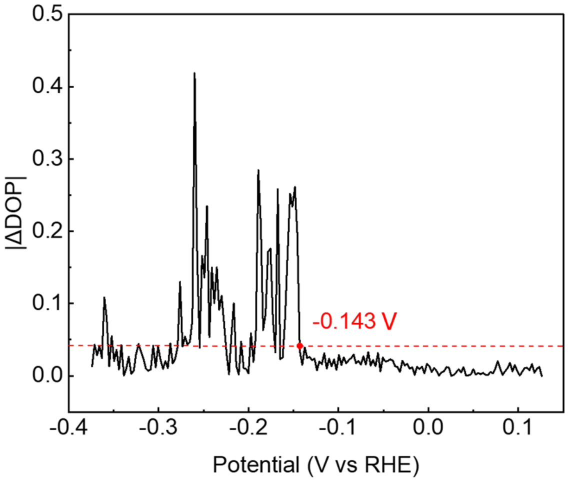

Fig. S37 |ΔDOP|-potential curve. The relationship between |ΔDOP| and applied potential on one pixel during LSV test of the Ni foam. The potential of -0.143 V vs RHE is defined as the onset potential of hydrogen evolution reaction of the pixel.

Supplementary Table

Table S1. Synthesis conditions of HMHs.

	Materials	Metal foam	Precursor	Temperature (°C)	Time (h)	Environment
$A\text{-}AB_8X_8$	$Co\text{-}Co_9S_8$	Co	S	700	4	Vacuum
	$CoNi\text{-}Co_9S_8$	Co	NiS_2	750	4	Vacuum
	$Co\text{-}NiCo_8S_8$	Co	NiS_2	700	4	Vacuum
	$Co\text{-}Co_8FeS_8$	Co	FeS_2	700	4	Vacuum
$A\text{-}AB_2X_4$	$Fe\text{-}FeMo_2S_4$	Fe	MoS_2	750	8	Vacuum
	$Ni\text{-}FeNi_2S_4$	Ni	FeS_2	700	4	Vacuum
	$Co\text{-}CoMo_2S_4$	Co	MoS_2	780	40	Vacuum
	$Co\text{-}NiCo_2Se_4$	Co	$NiSe_2$	700	4	Vacuum
	$Co\text{-}CoFe_2Se_4$	Co	$FeSe_2$	700	4	Vacuum
	$Cu\text{-}CuMo_2S_3$	Cu	MoS_2	750	1	Ar
$A\text{-}AB_6X_8$	$Cu\text{-}CuMo_6S_8$	Cu	MoS_2	750	1	Ar/H_2
	$Fe\text{-}FeMo_6S_8$	Fe	Mo:S (6:10)	800	10	Vacuum
	$Cu\text{-}CuMo_6Se_8$	Cu	$MoSe_2$	850	5	Ar
	$Fe\text{-}FeMo_6Se_8$	Fe	$MoSe_2$	950	8	Vacuum
$A\text{-}A_mX_n$	Fe-FeS	Fe	S	700	4	Vacuum
	$Ni\text{-}Ni_3S_2$	Ni	S	700	4	Vacuum
	$NiFe\text{-}Ni_3Se_2$	Ni	$FeSe_2$	700	4	Vacuum
	$CoMo\text{-}Mo_{15}Se_{19}$	Co	$MoSe_2$	850	20	Vacuum
	Fe-FeSe	Fe	Se	700	4	Vacuum
	$Co\text{-}Co_3Se_4$	Co	Se	950	4	Vacuum

Table S2. Electrochemical parameters of HMHs and references.

Number	R_{ct} (Ω)	$\Delta\eta/\Delta\log\|j\|$@ 1-2 A cm^{-2}	η@ 2 A cm^{-2}	Materials	η@ 2 A cm^{-2}	$\Delta\eta/\Delta\log\|j\|$@ 1-2 A cm^{-2}	R_{ct} (Ω)
1	1.46	284	352	Co-Co_8FeS_8	350	108	0.92
2	2.56	180	364	Co-$NiCo_8S_8$	422	94	1.33
3	1.15	217	453	Co-Co_9S_8	501	181	0.73
4	0.38	329	568	CoNi-Co_9S_8	522	378	0.28
5	1.27	44	336	Cu-$CuMo_6S_8$	-	-	-
6	1.41	101	483	Cu-$CuMo_2S_3$	-	-	-
7	2.26	45	367	Fe-$FeMo_6S_8$	440	80	1.18
8	1.01	237	577	Cu-$CuMo_6Se_8$	-	-	-
9	1.28	264	566	Fe-$FeMo_6Se_8$	530	332	1.05
10	1.56	96	371	Co-$CoMo_2S_4$	450	120	0.53
11	-	93	525	Ni-$FeNi_2S_4$	439	72	1.82
12	3.43	340	441	Fe-$FeMo_2S_4$	502	213	1.68
13	1.06	253	626	Ni-$NiCo_2Se_4$	541	340	0.72
14	-	66	524	Fe-$CoFe_2Se_4$	508	349	1.03
15	1.10	164	493	Ni-Ni_3S_2	384	22	0.23
16	0.33	386	596	Fe-FeS	492	256	0.89
17	0.52	365	603	Fe-FeSe	480	216	0.55
18	3.26	186	569	Mo-$Mo_{15}Se_{19}$	478	163	0.85
19	1.68	246	650	Ni-Ni_3Se_2	353	164	0.45
20	2.49	178	591	Co-Co_3Se_4	455	249	0.39
	7.40	202	522	Reference	630	387	2.30

Table S3. Optical and electrochemical screening results of CoFe-HMH.

Samples	Loading (mg cm^{-2})	Temp. (°C)	Vacuum (Pa)	Optical η (mV)	Electrochemical η (mV)
1	10	950	0.001	274.8563	276
2	10	950	0.1	267.9863	271
3	10	950	1	302.6919	305
4	10	750	0.001	260.4404	263
5	10	750	0.1	256.0352	261
6	10	750	1	305.5227	308
7	10	550	0.001	310.0964	289
8	10	550	0.1	259.2945	307
9	10	550	1	305.2807	311
10	5	950	0.001	264.0155	261
11	5	950	0.1	295.5444	264
12	5	950	1	296.7554	308
13	5	750	0.001	235.0375	246
14	5	750	0.1	261.8265	267
15	5	750	1	305.1329	307
16	5	550	0.001	315.3377	306
17	5	550	0.1	291.6248	302
18	5	550	1	321.5808	316
19	1	950	0.001	303.9055	293
20	1	950	0.1	301.5784	314
21	1	950	1	319.7608	359
22	1	750	0.001	329.0498	296
23	1	750	0.1	312.3773	321
24	1	750	1	356.3071	334
25	1	550	0.001	294.5878	312
26	1	550	0.1	343.8897	342
27	1	550	1	367.4340	362

Table S4. Comparison of HER performance between CuMo-HMH and other reported non-noble metal catalysts at 2 A cm^{-2}.

Catalysts	η (mV) @ 2 A cm^{-2}	Refs
$Nb_{1.35}S_2$	360	5
Ni_2P/NF	417	6
α-MoB_2	413	7
Nano-KFO/NF	340	8
F-Co_2P/Fe_2P/IF	300	9
Ta-TaS_2	398	10
**CuMo-HMH**	**336**	**This work**

Table S5. Comparison of OER performance between CoFe-HMH and other reported non-noble metal catalysts at 2 A cm^{-2}.

Catalysts	η (mV) @ 2 A cm^{-2}	Refs
$Ni_{78}Fe_{22}$-P	327	11
$Ni_{78}Fe_{22}$	415	11
$Ni_{80}Fe_{20}$	450	11
$Ni_{85}Fe_{15}$	510	11
Co-P-B-NF	430	12
**CoFe-HMH**	**350**	**This work**

Table S6. Comparison of AEMWE stability between HMHs and other reported devices running for over hundreds of hours.

Catalysts	Current density (mA cm^{-2})	Temperature (°C)	Electrolyte	Decay rate (mV h^{-1})	Refs
CAPist-L1	1000	25	1 M KOH	0.035	13
LFA	2000	50	1 M KOH	0.11	14
NiFe LDH	1000	60	Pure water	0.16	15
N-CoO	1000	60	1 M KOH	0.797	16
Bi/BiCeO$_{1.8}$H	1000	50	1 M KOH	0.40	17
RuGa SA/N-C	1000	60	1 M KOH	0.0497	18
NiFeO$_x$	1000	70	1 M KOH	0.228	19
Ni(Fe) MOF	500	60	1 M KOH	0.267	20
RuZn-Co_3O_4	500	60	1 M KOH	1.3	21
NiFe LDH	1000	60	1 M KOH	0.6	22
**HMHs**	500	25	**1 M KOH**	**0.00106**	**This work**

Table S7. Volume parameter for Co-Co_8FeS_8, Cu-$CuMo_6S_8$, Co-$CoMo_2S_4$ HMHs and corresponding mismatch degrees.

**Structure**	**Component A/B**	**n_a**	**n_b**	**Mismatch a**	**Mismatch b**
`	$FeCo_8S_8$ (1 1 1)	1	1	1.0 %	1.0 %
	Co (1 1 1)	3	3		
Cu-$CuMo_6S_8$	$CuMo_6S_8$ (0 1 2)`	2	2	2.3 %	3.6 %
	Cu (1 1 1)	5	9		
Co-$CoMo_2S_4$	$CoMo_2S_4$ (1 1 1)	4	3	0.4 %	1.0 %
	Co (1 1 1)	5	4		

References

1. Kresse G and Joubert D. From ultrasoft pseudopotentials to the projector augmented-wave method. *Phys Rev B* 1999; **59**: 1758-75.
2. Wang L, Maxisch T and Ceder G. Oxidation energies of transition metal oxides within the GGA+U framework. *Phys Rev B* 2006; **73**: 195107.
3. Pham HH, Barkema GT and Wang LW. DFT+U studies of Cu doping and p-type compensation in crystalline and amorphous ZnS. *Phys Chem Chem Phys* 2015; **17**: 26270-6.
4. Jain A, Hautier G, Ong SP, *et al*. Formation enthalpies by mixing GGA and GGA+U calculations. *Phys Rev B* 2011; **84**: 045115.
5. Yang J, Mohmad AR, Wang Y, *et al*. Ultrahigh-current-density niobium disulfide catalysts for hydrogen evolution. *Nat Mater* 2019; **18**: 1309-14.
6. Yu X, Yu ZY, Zhang XL, *et al*. "Superaerophobic" nickel phosphide nanoarray catalyst for efficient hydrogen evolution at ultrahigh current densities. *J Am Chem Soc* 2019; **141**: 7537-43.
7. Chen Y, Yu G, Chen W, *et al*. Highly active, nonprecious electrocatalyst comprising borophene subunits for the hydrogen evolution reaction. *J Am Chem Soc* 2017; **139**: 12370-3.
8. Jian J, Chen W, Zeng D, *et al*. Metal-ionic-conductor potassium ferrite nanocrystals with intrinsic superhydrophilic surfaces for electrocatalytic water splitting at ultrahigh current densities. *J Mater Chem A* 2021; **9**: 7586-93.
9. Zhang XY, Zhu YR, Chen Y, *et al*. Hydrogen evolution under large-current-density based on fluorine-doped cobalt-iron phosphides. *Chem Eng J* 2020; **399**: 125831.
10. Yu Q. Zhang Z, Qiu S, *et al*. A Ta-TaS_2 monolith catalyst with robust and metallic interface for superior hydrogen evolution. *Nat Commun* 2021; **12**: 6051.
11. Nairan A, Feng Z, Zheng R, *et al*. Engineering metallic alloy electrode for robust and active water electrocatalysis with large current density exceeding 2000 mA cm^{-2}. *Adv Mater* 2024; **36**: e2401448.
12. Silviya R, Bhide A, Gupta S, *et al*. Bifunctional amorphous transition-metal

phospho-boride electrocatalysts for selective alkaline seawater splitting at a current density of 2 A cm^{-2}. *Small Methods*, 2024; e2301395.

13. Li Z, Lin G, Wang L, *et al.* Seed-assisted formation of NiFe anode catalysts for an ion exchange membrane water electrolysis at industrial-scale current density. *Nat Catal* 2024; **7**: 944-52.14.
14. Wang J, Liang C, Ma X, *et al.* Dynamically adaptive bubbling for upgrading oxygen evolution reaction using lamellar fern-like alloy aerogel self-standing electrodes. *Adv Mater* 2024; **36**: e2307925.
15. Wan L, Liu J, Lin D, *et al.* 3D-ordered catalytic nanoarrays interlocked on anion exchange membranes for water electrolysis. *Energy Environ Sci* 2024; **17**, 3396-408.
16. Yu P., Zhang XL, Zhang TY, *et al.* Nitrogen-mediated promotion of cobalt-based oxygen evolution catalyst for practical anion-exchange membrane electrolysis. *J Am Chem Soc* 2024; **146**, 20379.
17. Seunghwan J. Jeon JI, Shin KH, *et al.* Stabilization of lattice oxygen evolution reactions in oxophilic Ce-mediated Bi/$BiCeO_{1.8}H$ electrocatalysts for efficient anion exchange membrane water electrolyzers. *Adv Mater* 2024; 2314211.
18. Zhou C, Shi J, Dong Z, *et al.* Oxophilic gallium single atoms bridged ruthenium clusters for practical anion-exchange membrane electrolyzer. *Nat Commun* 2024; **15**: 6741.
19. Yao R, Sun K, Zhang K, *et al.* Stable hydrogen evolution reaction at high current densities via designing the Ni single atoms and Ru nanoparticles linked by carbon bridges. *Nat Commun* 2024; **15**: 2218.
20. Li Y, Yang L, Hao X, *et al.* Origin of enhanced oxygen evolution in restructured metal-organic frameworks for anion exchange membrane water electrolysis. *Angew Chem Int Edit* 2024; e202413916.
21. Zhang G, Pei J, Wang Y, *et al.* Selective activation of lattice oxygen site through coordination engineering to boost the activity and stability of oxygen evolution reaction. *Angew Chem Int Edit* 2024; e202407509.
22. Klingenhof M, Trzesniowski H, Koch S, *et al.* High-performance anion-exchange

membrane water electrolysers using NiX (X= Fe, Co, Mn) catalyst-coated membranes with redox-active Ni-O ligands. *Nat Catal* 2024; **7:** 1213-22.
